# Supplementary material for: Deep learning integrates histopathology and proteogenomics at a pan-cancer level
Source: Cell Rep Med. 2023 Aug 14;4(9):101173. doi: 10.1016/j.xcrm.2023.101173 (PMC10518635; doi:10.1016/j.xcrm.2023.101173)
Supplement: Document S1. Figures S1–S6 [file mmc1.pdf]

Cell Reports Medicine, Volume *4*

Supplemental information

Deep learning integrates histopathology and proteogenomics at a pan-cancer level

Joshua M. Wang, Runyu Hong, Elizabeth G. Demicco, Jimin Tan, Rossana Lazcano, Andre L. Moreira, Yize Li, Anna Calinawan, Narges Razavian, Tobias Schraink, Michael A. Gillette, Gilbert S. Omenn, Eunkyung An, Henry Rodriguez, Aristotelis Tsirigos, Kelly V. Ruggles, Li Ding, Ana I. Robles, D.R. Mani, Karin D. Rodland, Alexander J. Lazar, Wenke Liu, David Fenyö, and Clinical Proteomic Tumor Analysis Consortium

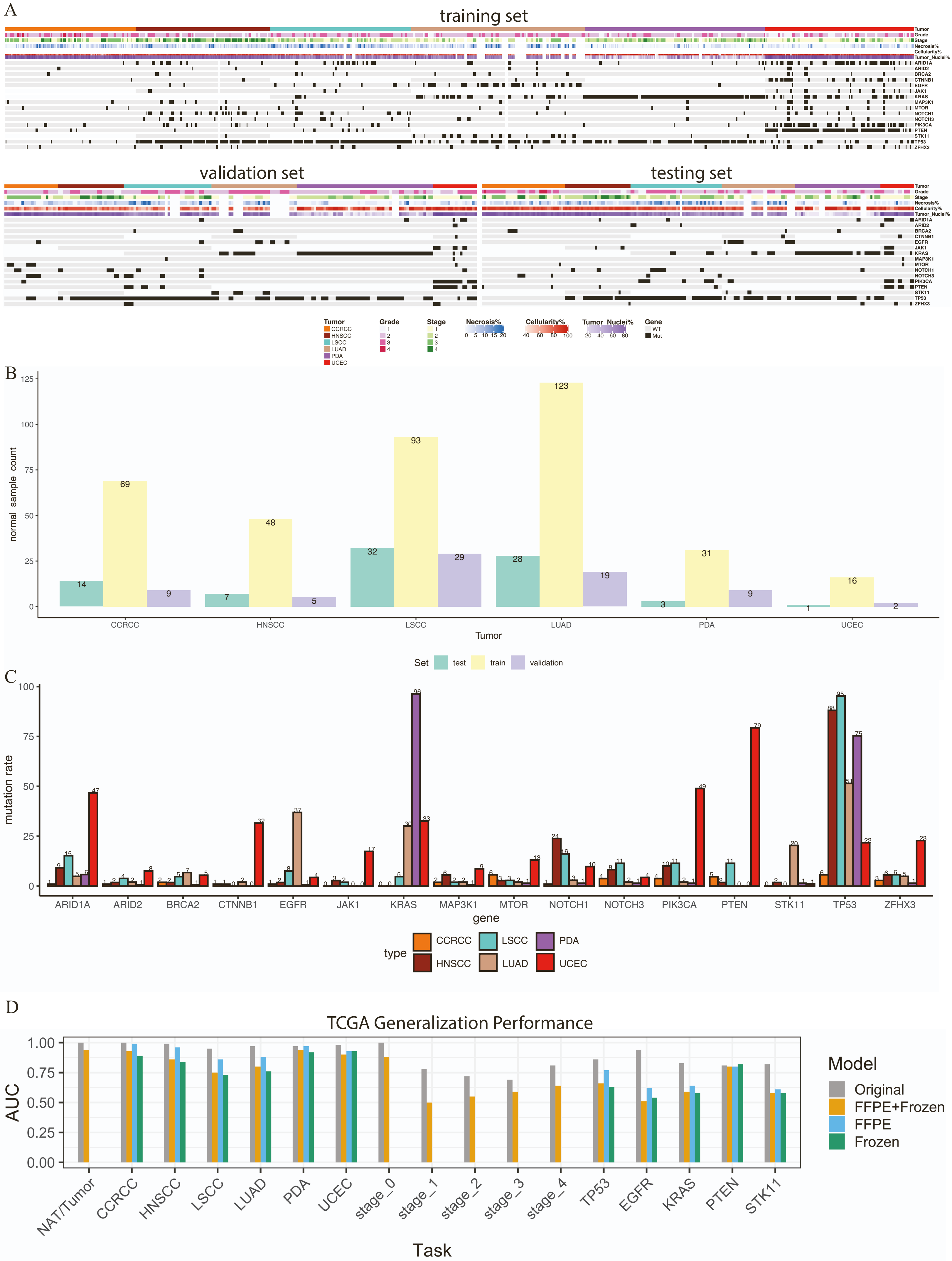

A
training set
validation set
testing set
Tumor
Grade
Stage
Necrosis%
Cellularity%
Tumor_Nuclei%
ARID1A
ARID2
BRCA2
CTNNB1
EGFR
JAK1
KRAS
MAP3K1
MTOR
NOTCH1
NOTCH3
PIK3CA
PTEN
STK11
TP53
ZFHX3
Tumor
CCRCC
HNSCC
LSCC
LUAD
PDA
UCEC
Grade
1
2
3
4
Stage
1
2
3
4
Necrosis%
0 5 10 15 20
Cellularity%
40 60 80 100
Tumor_Nuclei%
20 40 60 80
Gene
WT
Mut
B
normal_sample_count
Tumor
CCRCC
HNSCC
LSCC
LUAD
PDA
UCEC
Set
test
train
validation
C
mutation rate
gene
ARID1A
ARID2
BRCA2
CTNNB1
EGFR
JAK1
KRAS
MAP3K1
MTOR
NOTCH1
NOTCH3
PIK3CA
PTEN
STK11
TP53
ZFHX3
type
CCRCC
LSCC
PDA
HNSCC
LUAD
UCEC
D
TCGA Generalization Performance
AUC
Task
NAT/Tumor
CCRCC
HNSCC
LSCC
LUAD
PDA
UCEC
stage_0
stage_1
stage_2
stage_3
stage_4
TP53
EGFR
KRAS
PTEN
STK11
Model
Original
FFPE+Frozen
FFPE
Frozen

Figure S1: Data summary and generalization performance, related to Figure 1.

(A) Heatmaps show clinical and molecular features with 70:15:15 split at per-patient level for training, validation and testing sets.
(B) Number of normal slides in each set.
(C) Common gene mutation rates of samples in our cohort by cancer type.
(D) TCGA external validation for all imaging-based prediction tasks reported at per-slide AUROCs.

A

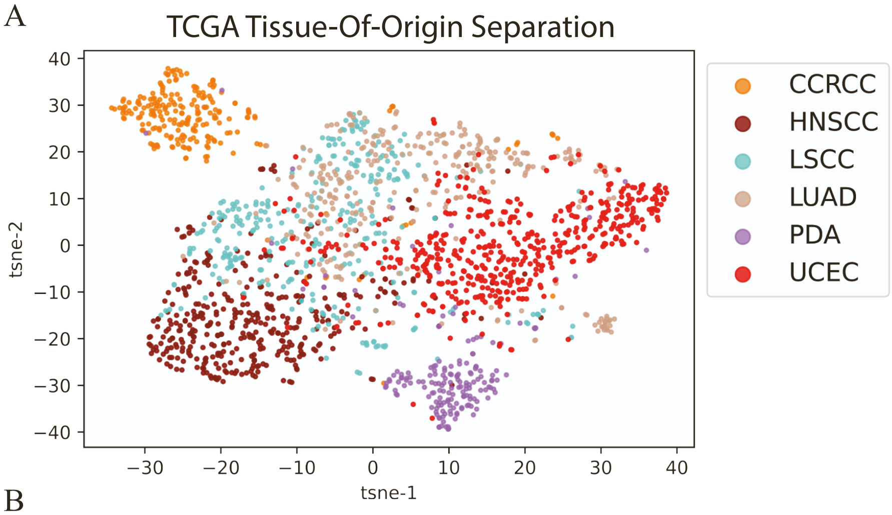

B

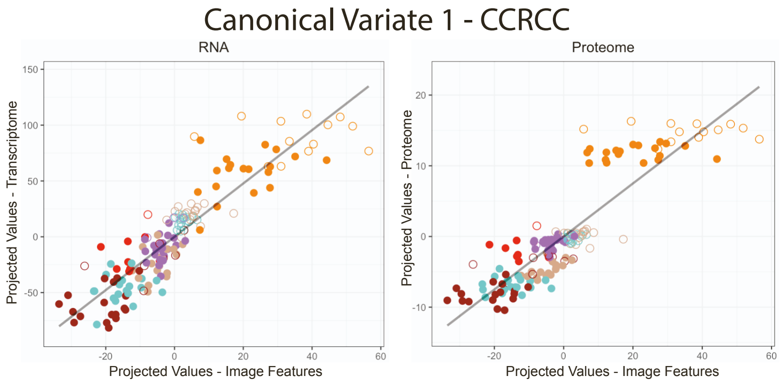

top GO terms (RNA)	q
fatty acid oxidation	1.5 e-18
alpha-amino acid catabolic process	6.9 e-11
peroxisome organization	1.6 e-10

bottom GO terms (RNA)	q
regulation of chromosome segregation	4.1 e-13
cornification	1.1 e-10
keratinization	1.2 e-10

top GO terms (Proteome)	q
aromatic amino acid family catabolic process	1.0 e-2
endothelium development	1.1 e-2
actin filament capping	1.1 e-2

bottom GO terms (Proteome)	q
epidermis morphogenesis	8.8 e-4
regulation of hair cycle	1.1 e-3
intermediate filament organization	1.2 e-3

C

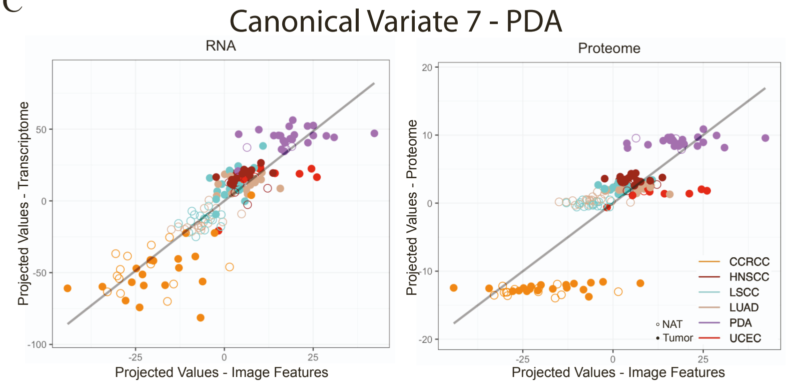

top GO terms (RNA)	q
collagen metabolic process	1.5 e-6
SRP-dependent cotranslational protein targeting to membrane	4.6 e-6
extracellular matrix disassembly	1.8 e-4

bottom GO terms (RNA)	q
alpha-amino acid catabolic process	4.4 e-9
dicarboxylic acid metabolic process	1.5 e-7
cofactor catabolic process	2.5 e-4

top GO terms (Proteome)	q
regulation of epithelial cell apoptotic process	4.4 e-3
regulation of response to wounding	4.7 e-3
negative regulation of Wnt signaling pathway	4.7 e-3

D

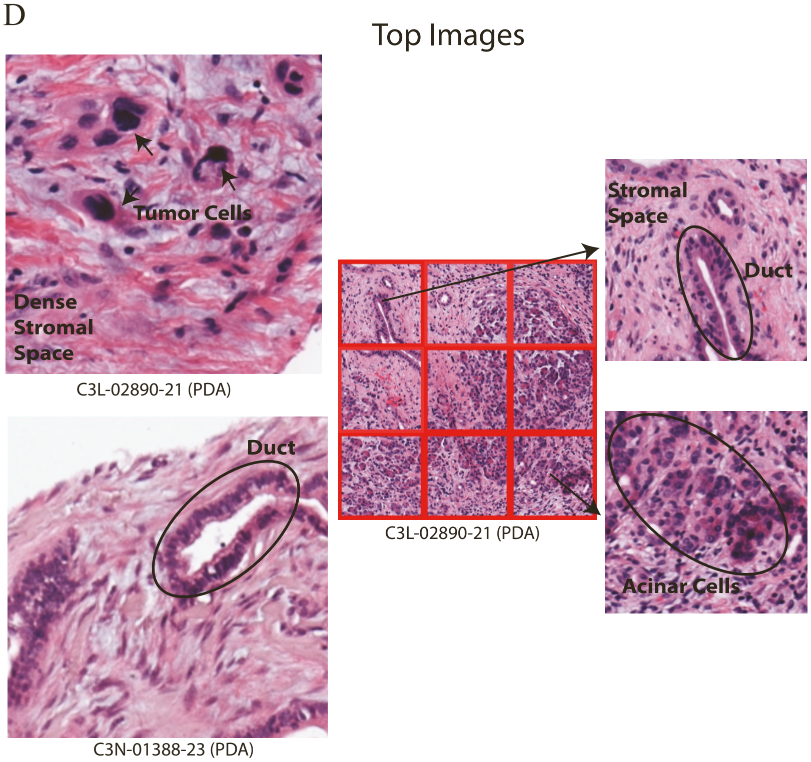

Figure S2: Additional covariates explaining tissue-of-origin imaging features, related to Figure 2.

(A) TSNE separation of latent features from external TCGA test set.
(B) Strongest covariate distinctly separates CCRCC from all others. Top and bottom GO terms explain the enrichment of genes/proteins selected to separate images into top and bottom scores respectively.
(C) Top terms correspond to enrichment of genes/proteins separating PDA samples from others.
(D) Top scoring images exhibit morphologies consistent with selected proteogenomic features.

A

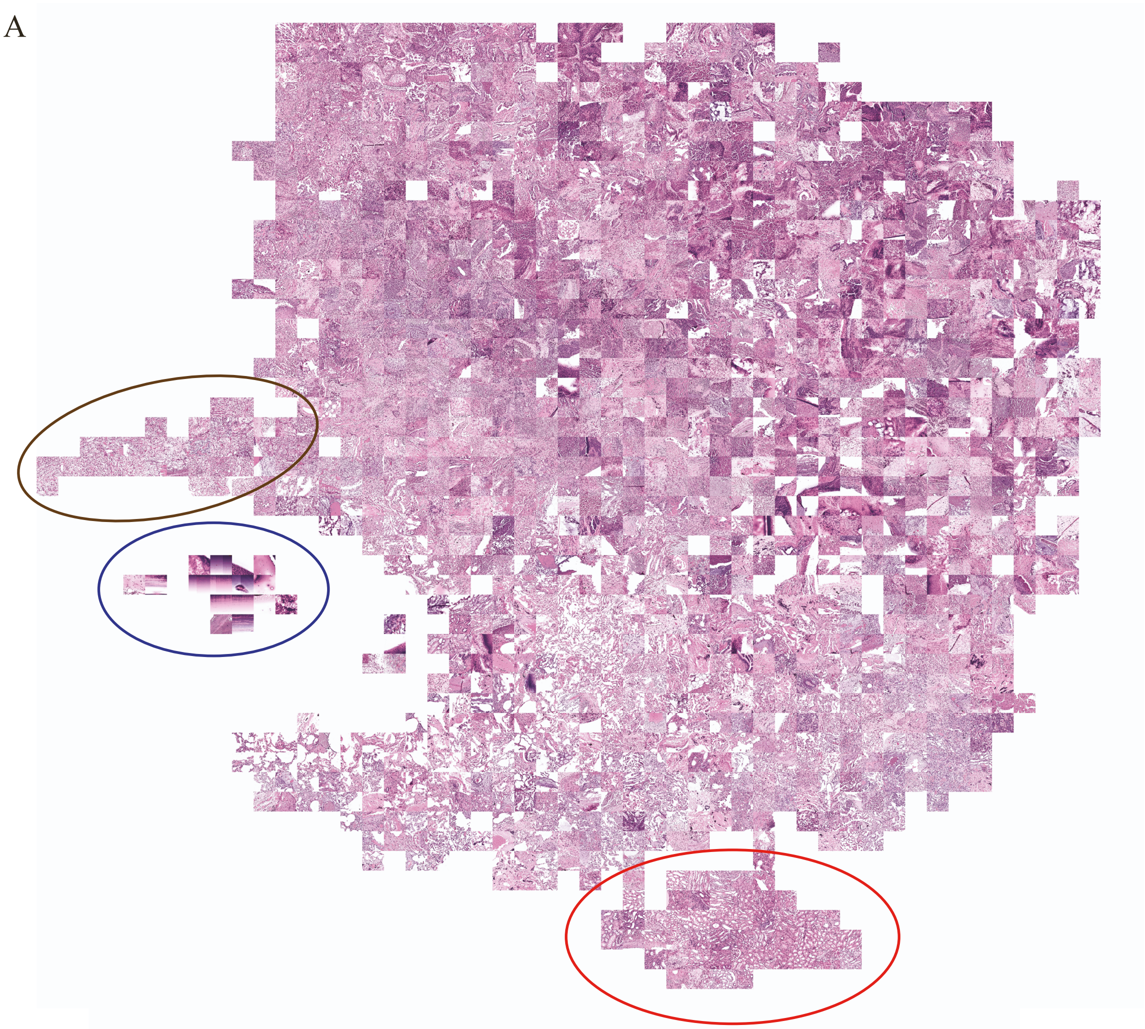

B

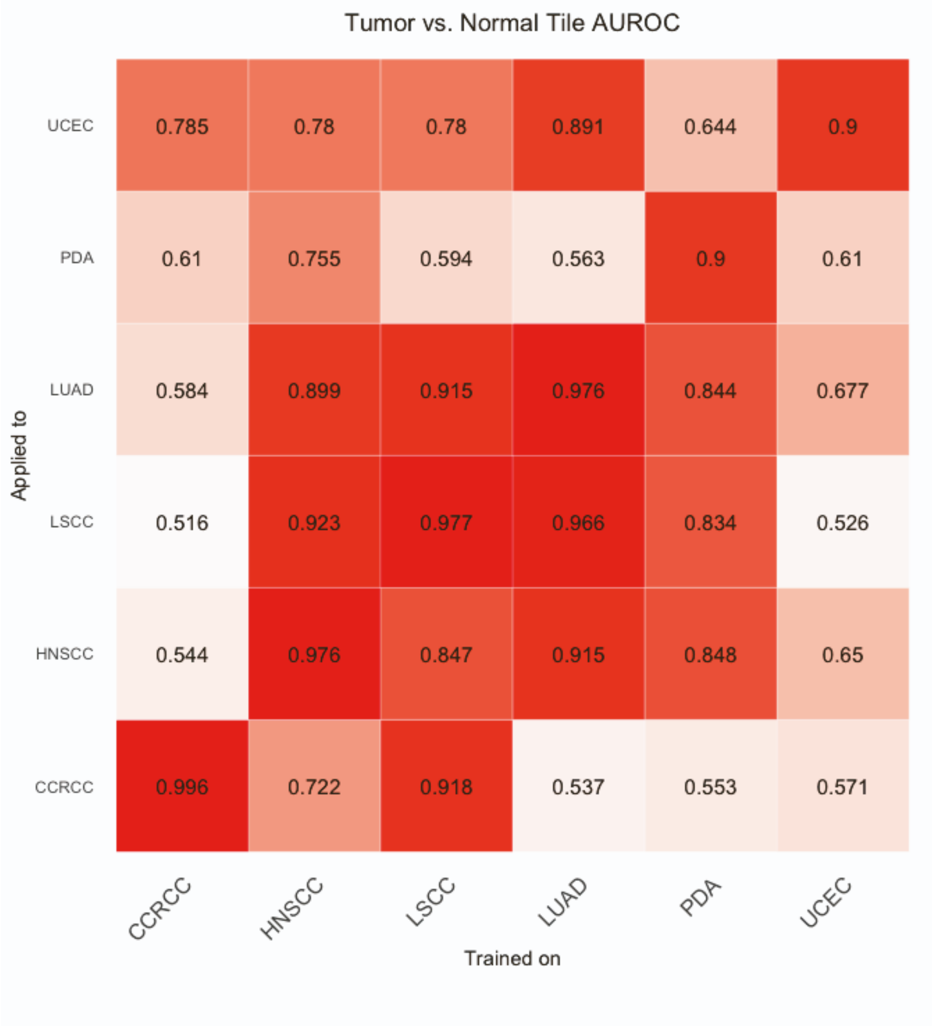
Tumor vs. Normal Tile AUROC
Applied to
UCEC
0.785
0.78
0.78
0.891
0.644
0.9
PDA
0.61
0.755
0.594
0.563
0.9
0.61
LUAD
0.584
0.899
0.915
0.976
0.844
0.677
LSCC
0.516
0.923
0.977
0.966
0.834
0.526
HNSCC
0.544
0.976
0.847
0.915
0.848
0.65
CCRCC
0.996
0.722
0.918
0.537
0.553
0.571
CCRCC
HNSCC
LSCC
LUAD
PDA
UCEC
Trained on

Figure S3: Detailed feature visualization of tumorigenesis model and per-tile level cross testing performance, related to Figure 3.

(A) Extraction of features learned by pan-cancer tumorigenesis model. Brown circle highlighting distinctive CCRCC features. Red circle highlighting non-neoplastic renal tubules. Blue circle highlighting artifacts.

(B) Heatmap showing the per-tile level AUROCs of applying single cancer type trained models to the other cancer type samples.

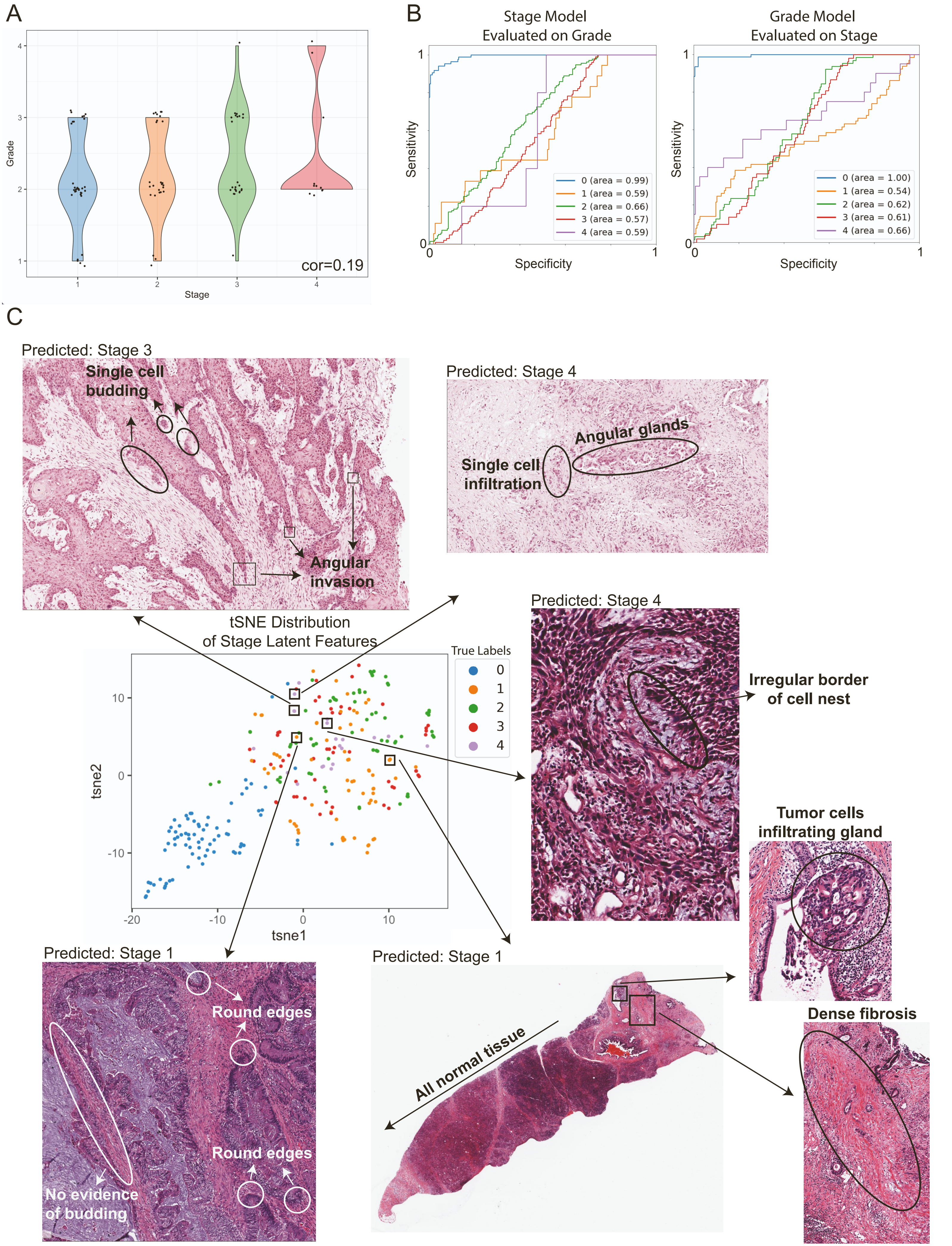

A
Grade
Stage
cor=0.19
B
Stage Model
Evaluated on Grade
Grade Model
Evaluated on Stage
Sensitivity
Specificity
0 (area = 0.99)
1 (area = 0.59)
2 (area = 0.66)
3 (area = 0.57)
4 (area = 0.59)
0 (area = 1.00)
1 (area = 0.54)
2 (area = 0.62)
3 (area = 0.61)
4 (area = 0.66)
C
Predicted: Stage 3
Single cell budding
Angular invasion
Predicted: Stage 4
Angular glands
Single cell infiltration
tSNE Distribution of Stage Latent Features
True Labels
tsne1
tsne2
Predicted: Stage 4
Irregular border of cell nest
Tumor cells infiltrating gland
Predicted: Stage 1
Round edges
Round edges
No evidence of budding
Predicted: Stage 1
All normal tissue
Dense fibrosis

Figure S4: Slide level performance of percent nuclei, necrosis, and cellularity, related to Figure 5.

(A) CPTAC grade and stage labels do not correlate.
(B) Models trained to predict grade and stage do not generalize to predicting the other label.
(C) Manual histology review of differing features amongst samples predicted at varying degrees of tumor stage.

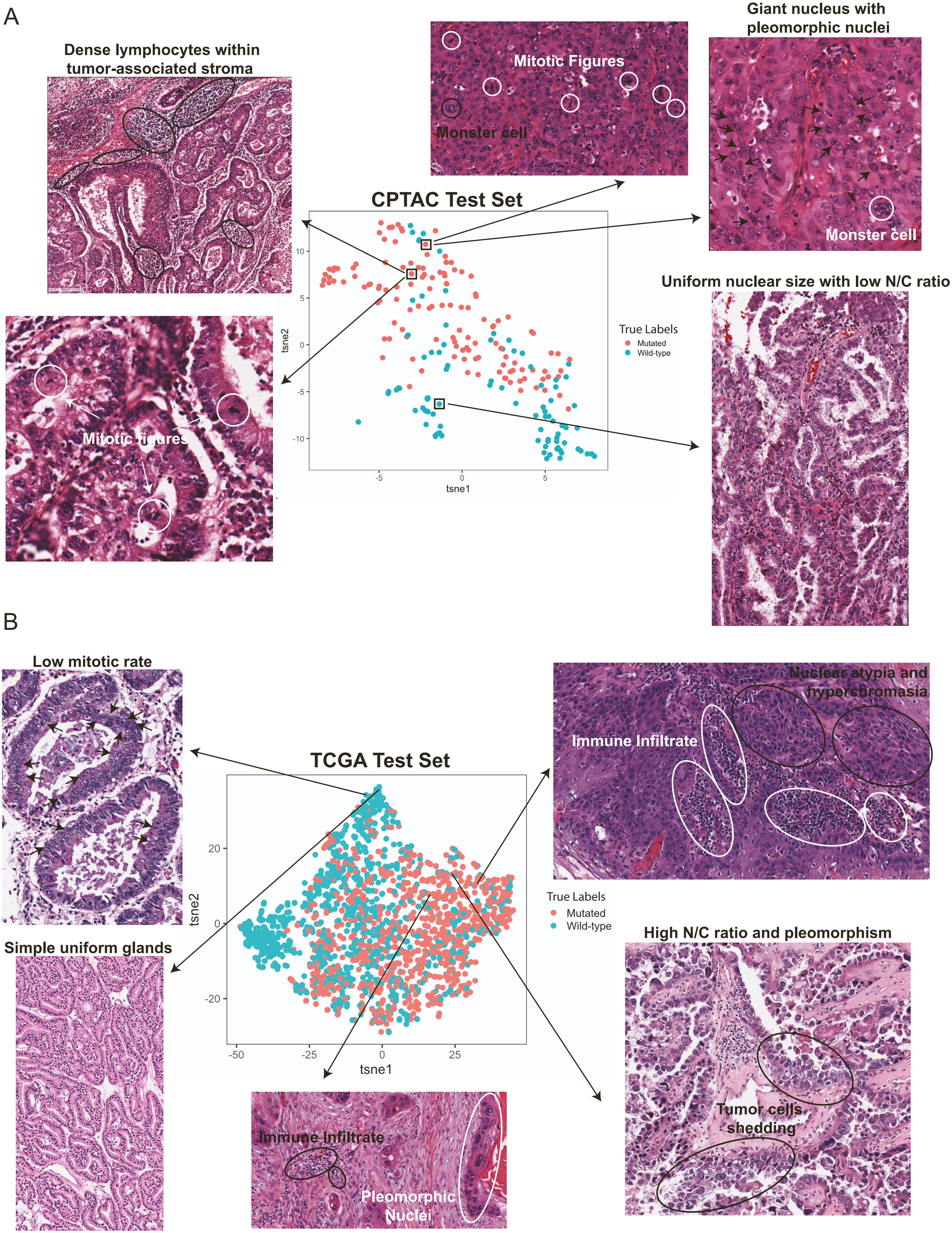
A
Dense lymphocytes within tumor-associated stroma
Mitotic Figures
Monster cell
Giant nucleus with pleomorphic nuclei
Monster cell
CPTAC Test Set
tsne2
tsne1
10
5
0
-5
-10
-5
0
5
True Labels
Mutated
Wild-type
Uniform nuclear size with low N/C ratio
Mitotic figures
B
Low mitotic rate
TCGA Test Set
Nuclear atypia and hyperchromasia
Immune Infiltrate
tsne2
tsne1
20
0
-20
-50
-25
0
25
True Labels
Mutated
Wild-type
Simple uniform glands
High N/C ratio and pleomorphism
Tumor cells shedding
Immune Infiltrate
Pleomorphic Nuclei

Figure S5: Visualization of features learned by the TP53 model for internal and external test sets, related to Figure 6.

(A) CPTAC.

(B) TCGA.

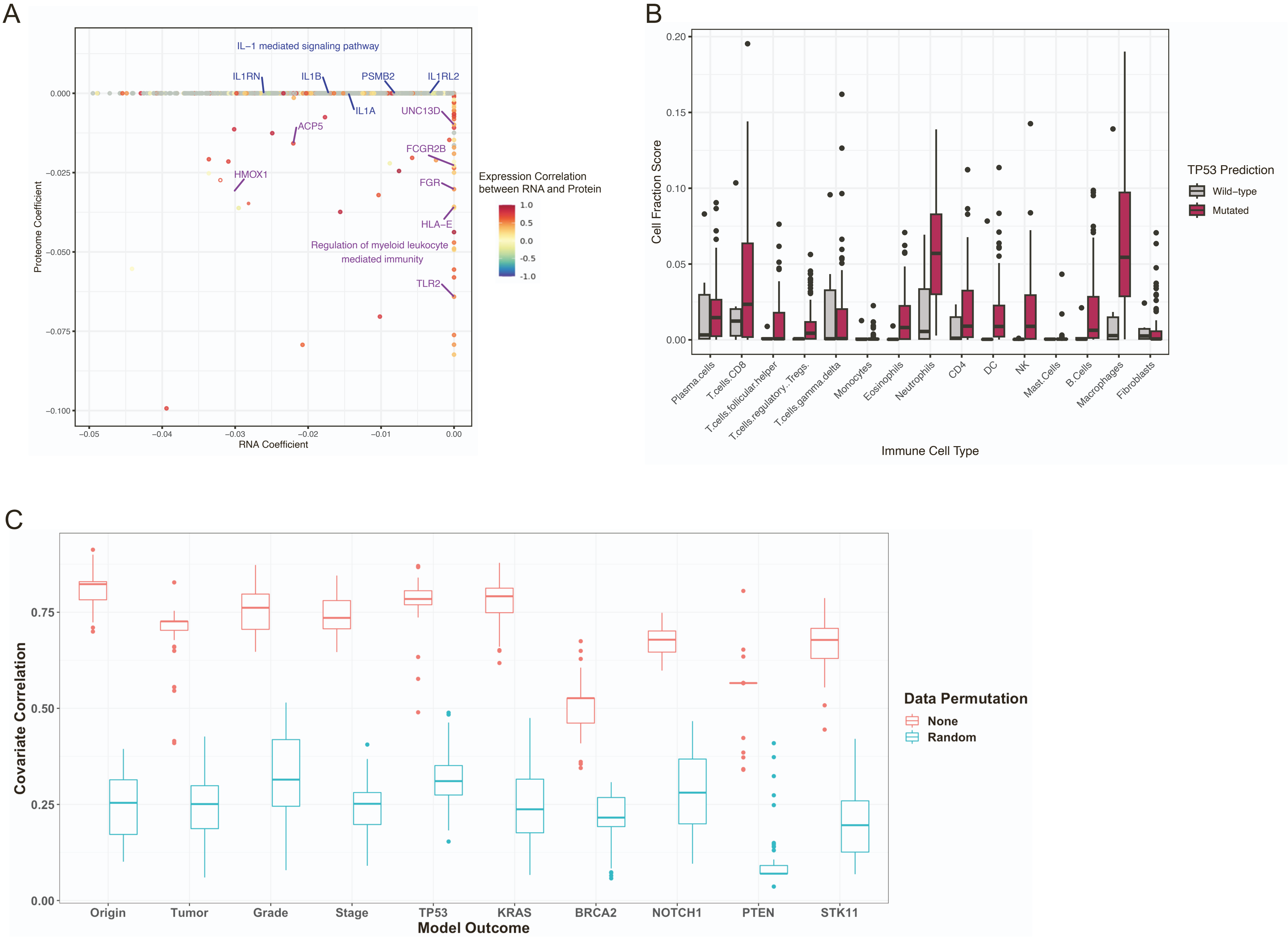
A
IL–1 mediated signaling pathway
IL1RN
IL1B
PSMB2
IL1RL2
IL1A
UNC13D
ACP5
FCGR2B
HMOX1
FGR
HLA–E
Regulation of myeloid leukocyte mediated immunity
TLR2
Proteome Coefficient
RNA Coefficient
0.000
−0.025
−0.050
−0.075
−0.100
−0.05
−0.04
−0.03
−0.02
−0.01
0.00
Expression Correlation between RNA and Protein
1.0
0.5
0.0
-0.5
-1.0
B
Cell Fraction Score
0.20
0.15
0.10
0.05
0.00
TP53 Prediction
Wild–type
Mutated
Plasma.cells
T.cells.CD8
T.cells.follicular.helper
T.cells.regulatory..Tregs.
T.cells.gamma.delta
Monocytes
Eosinophils
Neutrophils
CD4
DC
NK
Mast.Cells
B.Cells
Macrophages
Fibroblasts
Immune Cell Type
C
Covariate Correlation
0.75
0.50
0.25
0.00
Origin
Tumor
Grade
Stage
TP53
KRAS
BRCA2
NOTCH1
PTEN
STK11
Model Outcome
Data Permutation
None
Random

Figure S6: Paired imaging-proteogenomic were randomly permuted to assess the baseline correlation detectable due to noise, related to Figure 6.

(A) Plot of the coefficients of each gene at the RNA and protein level. The dot color represents the expression correlation at RNA and protein level. Genes selected as important at the RNA level but not proteome will have a zero value on the y-axis but a non-zero value on the x-axis, and vice versa for those correlated at the proteome level but not RNA.

(B) Boxplot distribution of immune cell fractions calculated from BayesDeBulk proteogenomic deconvolution in TP53-mutated and wildtype test samples.

(C) Across all model outcomes, canonical variate correlations are significantly higher than that from permutation, suggesting that the additional correlation captured corresponds to genuine biological signals.
