# Supplementary material for: Deep learning integrates histopathology and proteogenomics at a pan-cancer level
Source: Cell Rep Med. 2023 Aug 14;4(9):101173. doi: 10.1016/j.xcrm.2023.101173 (PMC10518635; doi:10.1016/j.xcrm.2023.101173)
Supplement: Document S2. Article plus supplemental information [file mmc2.pdf]

Cell Reports Medicine

Article

Deep learning integrates histopathology and proteogenomics at a pan-cancer level

Graphical abstract

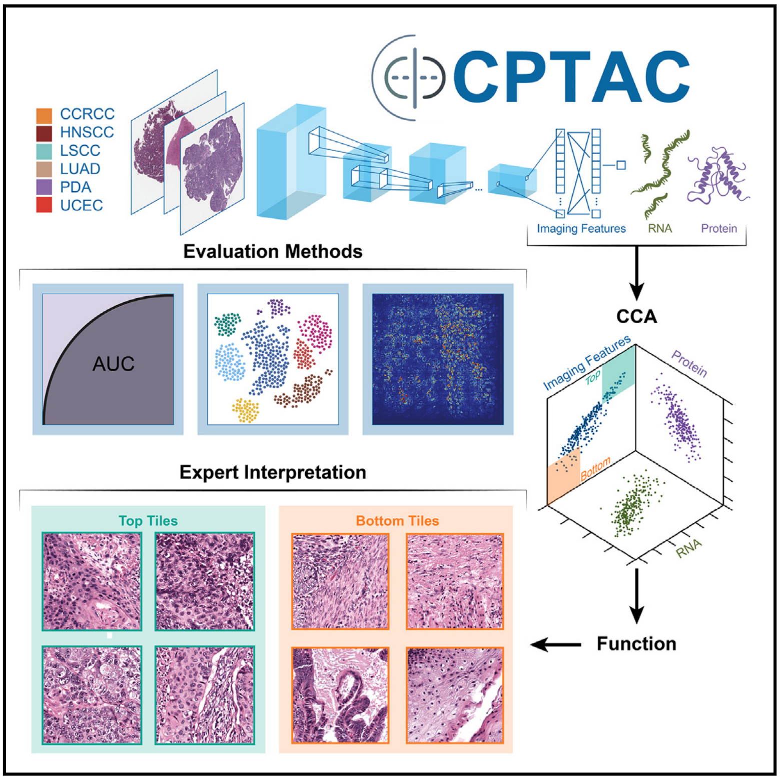

Authors

Joshua M. Wang, Runyu Hong, Elizabeth G. Demicco, ..., Wenke Liu, David Fenyö, Clinical Proteomic Tumor Analysis Consortium

Correspondence

alazar@mdanderson.org (A.J.L.),
wenke.liu@nyulangone.org (W.L.),
david@fenyolab.org (D.F.)

In brief

Wang et al. predict pan-cancer molecular outcomes from CPTAC H&E histology images and validate generalizability performance with TCGA. Transcriptomic and proteomics data are integrated to describe pathway-level molecular signature predictive of morphology features. Human experts identify clinically actionable histology features, a unique step not seen in prior studies.

Highlights

- Train and validate deep learning imaging models to pan-cancer proteogenomic data
- Image models identify diagnostic morphologies driving proteogenomic signatures
- Extensively integrate human expert review to improve interpretable visualization

Wang et al., 2023, Cell Reports Medicine *4*, 101173
September 19, 2023 © 2023 The Author(s).
https://doi.org/10.1016/j.xcrm.2023.101173

CellPress

Cell Reports Medicine

CellPress
OPEN ACCESS

Article

Deep learning integrates histopathology and proteogenomics at a pan-cancer level

Joshua M. Wang,[1,2,23] Runyu Hong,[1,2,23] Elizabeth G. Demicco,[3,24] Jimin Tan,[1,2,4,24] Rossana Lazcano,[5] Andre L. Moreira,[6] Yize Li,[7,8] Anna Calinawan,[9,10] Narges Razavian,[11,12] Tobias Schraink,[1,2,4] Michael A. Gillette,[13,14,15] Gilbert S. Omenn,[16] Eunkyung An,[17] Henry Rodriguez,[17] Aristotelis Tsirigos,[6,4] Kelly V. Ruggles,[1,4] Li Ding,[18,19] Ana I. Robles,[17] D.R. Mani,[13] Karin D. Rodland,[20,21] Alexander J. Lazar,[5,22,*] Wenke Liu,[1,2,*] David Fenyö,[1,2,25,*] and Clinical Proteomic Tumor Analysis Consortium

[1]Institute for Systems Genetics, NYU Grossman School of Medicine, New York, NY 10016, USA
[2]Department of Biochemistry and Molecular Pharmacology, NYU Grossman School of Medicine, New York, NY 10016, USA
[3]Department of Pathology and Laboratory Medicine, Mount Sinai Hospital and Laboratory Medicine and Pathobiology, University of Toronto, Toronto M5G 1X5, ON, Canada
[4]Division of Precision Medicine, Department of Medicine, NYU Grossman School of Medicine, New York, NY 10016, USA
[5]Department of Pathology, The University of Texas MD Anderson Cancer Center, Houston, TX 77030, USA
[6]Department of Pathology, NYU Grossman School of Medicine, New York, NY 10016, USA
[7]Department of Medicine, Washington University in St. Louis, St. Louis, MO 63110, USA
[8]McDonnell Genome Institute, Washington University in St. Louis, St. Louis, MO 63108, USA
[9]Department of Genetics and Genomic Sciences, Icahn School of Medicine at Mount Sinai, New York, NY 10029, USA
[10]Icahn Institute for Data Science and Genomic Technology, Icahn School of Medicine at Mount Sinai, New York, NY 10029, USA
[11]Department of Population Health, NYU Grossman School of Medicine, New York, NY 10016, USA
[12]Department of Radiology, NYU Grossman School of Medicine, New York, NY 10016, USA
[13]The Broad Institute of MIT and Harvard, Cambridge, MA 02142, USA
[14]Massachusetts General Hospital Division of Pulmonary and Critical Care Medicine, Boston, MA 02114, USA
[15]Harvard Medical School, Boston, MA 02115, USA
[16]Departments of Computational Medicine & Bioinformatics, Internal Medicine, Human Genetics, and School of Public Health, University of Michigan, Ann Arbor, MI 48109, USA
[17]Office of Cancer Clinical Proteomics Research, National Cancer Institute, Rockville, MD 20850, USA
[18]Department of Medicine and Genetics, Siteman Cancer Center, Washington University in St. Louis, St. Louis, MO 63110, USA
[19]McDonnell Genome Institute, Washington University in St. Louis, St. Louis, MO 63108, USA
[20]Biological Sciences Division, Pacific Northwest National Laboratory, Richland, WA 99354, USA
[21]Department of Cell, Developmental, and Cancer Biology, Oregon Health & Science University, Portland, OR 97221, USA
[22]Department of Genomic Medicine, The University of Texas MD Anderson Cancer Center, Houston, TX 77030, USA
[23]These authors contributed equally
[24]These authors contributed equally
[25]Lead contact
*Correspondence: alazar@mdanderson.org (A.J.L.), wenke.liu@nyulangone.org (W.L.), david@fenyolab.org (D.F.)
https://doi.org/10.1016/j.xcrm.2023.101173

SUMMARY

We introduce a pioneering approach that integrates pathology imaging with transcriptomics and proteomics to identify predictive histology features associated with critical clinical outcomes in cancer. We utilize 2,755 H&E-stained histopathological slides from 657 patients across 6 cancer types from CPTAC. Our models effectively recapitulate distinctions readily made by human pathologists: tumor vs. normal (AUROC = 0.995) and tissue-of-origin (AUROC = 0.979). We further investigate predictive power on tasks not normally performed from H&E alone, including TP53 prediction and pathologic stage. Importantly, we describe predictive morphologies not previously utilized in a clinical setting. The incorporation of transcriptomics and proteomics identifies pathway-level signatures and cellular processes driving predictive histology features. Model generalizability and interpretability is confirmed using TCGA. We propose a classification system for these tasks, and suggest potential clinical applications for this integrated human and machine learning approach. A publicly available web-based platform implements these models.

INTRODUCTION

Recent advances in machine learning inform precision medicine and translational research. Computational pathology applies computer vision methods to clinical and pathological images, and has benefited greatly from neural-network-based deep learning technologies.[1,2] Convolutional neural network (CNN) models can robustly predict commonly mutated genes in

This is an open access article under the CC BY license (http://creativecommons.org/licenses/by/4.0/).

specific cancer types, such as non-small cell lung cancer, where CNNs are able to predict six of the ten most commonly mutated genes with AUROCs ranging from 0.733 to 0.856.[3,4] We recently introduced a multi-resolution CNN architecture, Panoptes, to classify endometrial cancer images.[5] These techniques can be applied to cancer types from diverse organ systems, which can express similar molecular signatures despite their various origins.[6] Weakly supervised CNNs can also identify actionable driver mutations across multiple cancer types[7] and predict molecular features, including somatic mutation status such as *TP53* and *PTEN*, microsatellite instability, and molecular subtypes.[3,7,8] However, to our knowledge, there have been no prior attempts to correlate rich proteomic data with histologic features.

A major limitation of the field is that few weakly supervised deep learning studies have integrated pathology expertise into study design and interpretation, resulting in models that may lack human interpretability or clear relevance to tumor biology. Moreover, existing models rely heavily on genomic and transcriptomic data only, which do not always translate into biologically significant alterations in tumor proteome. Without validation of model transparency and identification of human-intuitive features correlating to model predictions, or validation of transcriptomic data at the protein level, real-world adoption of machine learning as an ancillary technique by clinical and translational researchers is severely limited.[9] Indeed, most studies attempting to correlate multidimensional datasets examine only one or two variables (e.g., mutation status and histology), and do not assess the impact of multi-omics data on human interpretability and relevance; nor do they define avenues to complement traditional histopathologic evaluation. To provide a conceptual framework addressing these problems, and provide a roadmap to increase uptake of the technology in clinical and research settings, we first introduce a classification scheme for computational tasks relative to the ability of humans to perform them. Class 1 tasks emulate what pathologists already perform, such as are seen with supervised training models, and class 2 tasks are those that CNNs are trained to perform that fall outside of pathologists' routine intuition. We then evaluate proteomic models to complement and validate transcriptomic correlations.

We utilized the Clinical Proteomic Tumor Analysis Consortium (CPTAC), which has comprehensively characterized tumor omics, ranging from genomics to proteomics and metabolomics, in hundreds of patients well-annotated with matching histopathology images and clinical outcomes.[10–17] This cohort has not yet been extensively mined for pan-cancer image analysis. The plethora of molecular and clinical data enables the construction of integrative and systematic molecular signatures, which can provide insight into mechanisms underlying a variety of cancers, as well as the workings of the CNN (Figure 1A). Critically, all image data from CPTAC represent mirror-image tissue sections immediately adjacent to the tissue subjected to omic analysis, allowing direct cognate correlation between molecular and imaging features. We further use canonical correlation analysis (CCA) to identify joint multivariate relationships between histology and omics data[18] and identify subsets of proteogenomic and imaging features that are biologically related.[19] This approach has been validated for other multi-omics datasets, including data from The Cancer Genome Atlas (TCGA),[20,21] to facilitate pathway-level knowledge discovery without leveraging prior biological understanding.

Our CNN models were trained with histopathological images from six different cancer types (clear cell renal cell carcinoma [CCRCC][11], head and neck squamous cell carcinoma [HNSCC][16], lung squamous cell carcinoma [LSCC][15], lung adenocarcinoma [LUAD][14], pancreatic ductal adenocarcinoma [PDA][17], and uterine corpus endometrial carcinoma [UCEC][13]), and were able to perform proof-of-concept class 1 tasks, such as distinguishing tumor from normal and tissue-of-origin, and more difficult class 2 tasks, such as identifying both tissue-specific morphological features related to clinical and biomarker features as well as pan-cancer tissue-level and cellular properties. For both task classes, we relate predictive morphologies to transcriptional and translational expression data to identify molecular signatures driving phenotype differences. Based on subsequent pathologist input, class 2 tasks were further subclassified. In class 2a, humans could intuit potential common histologic features within predicted groups, enabling them to learn potentially novel ways of viewing tumor classification For class 2b, histologic features separating predicted groups were not readily discernible to human pathologists. The integration and iteration of machine and human learning in class 2a tasks suggests new roles for synergistic machine learning tools in clinically relevant pathology evaluation.

Finally, we introduce a cloud-based graphical user interface for clinicians to apply our machine learning models to their own histological images. This workflow envisions a pathology-centered design where a single whole slide image (WSI) can be uploaded and computationally scored in the cloud. Generated heatmaps highlight regions of interest based on the selected predictive model.

RESULTS

Our image bank consisted of 2,755 H&E-stained slides sectioned from formalin-fixed paraffin-embedded (FFPE) tissue blocks obtained from the CPTAC including six cancer types: CCRCC, HNSCC, LSCC, LUAD, PDA, and UCEC, representing 2,217 total tumor tissue slides and 538 normal adjacent tissue (NAT) slides from 657 patients (Figure S1A). A total of 5,374 WSIs from TCGA was utilized for independent validation of model generalizability. Each WSI was divided into smaller tiles sharing the same label as the whole slide, and individual tile-level evaluations were then aggregated at whole-image level. Clinical attributes, histopathological features, gene mutations, and proteogenomic expressions for these samples were obtained from CPTAC[22] (Figures S1B and S1C) and TCGA as applicable. Panoptes-based multi-resolution CNN models were trained, validated, and tested on the tiles following the same data preparation protocol as in our previous publications.[5]

To better understand model decision-making, we categorized four tasks as class 1 or 2. Class 1 tasks were utilized as a proof-of-concept of the model and included (1) identifying tumor tissue-of-origin and (2) discriminating tumor from normal tissue. Class 2 tasks included prediction of (3) clinical features and (4) biomarkers from histologic image features.

CellPress
OPEN ACCESS

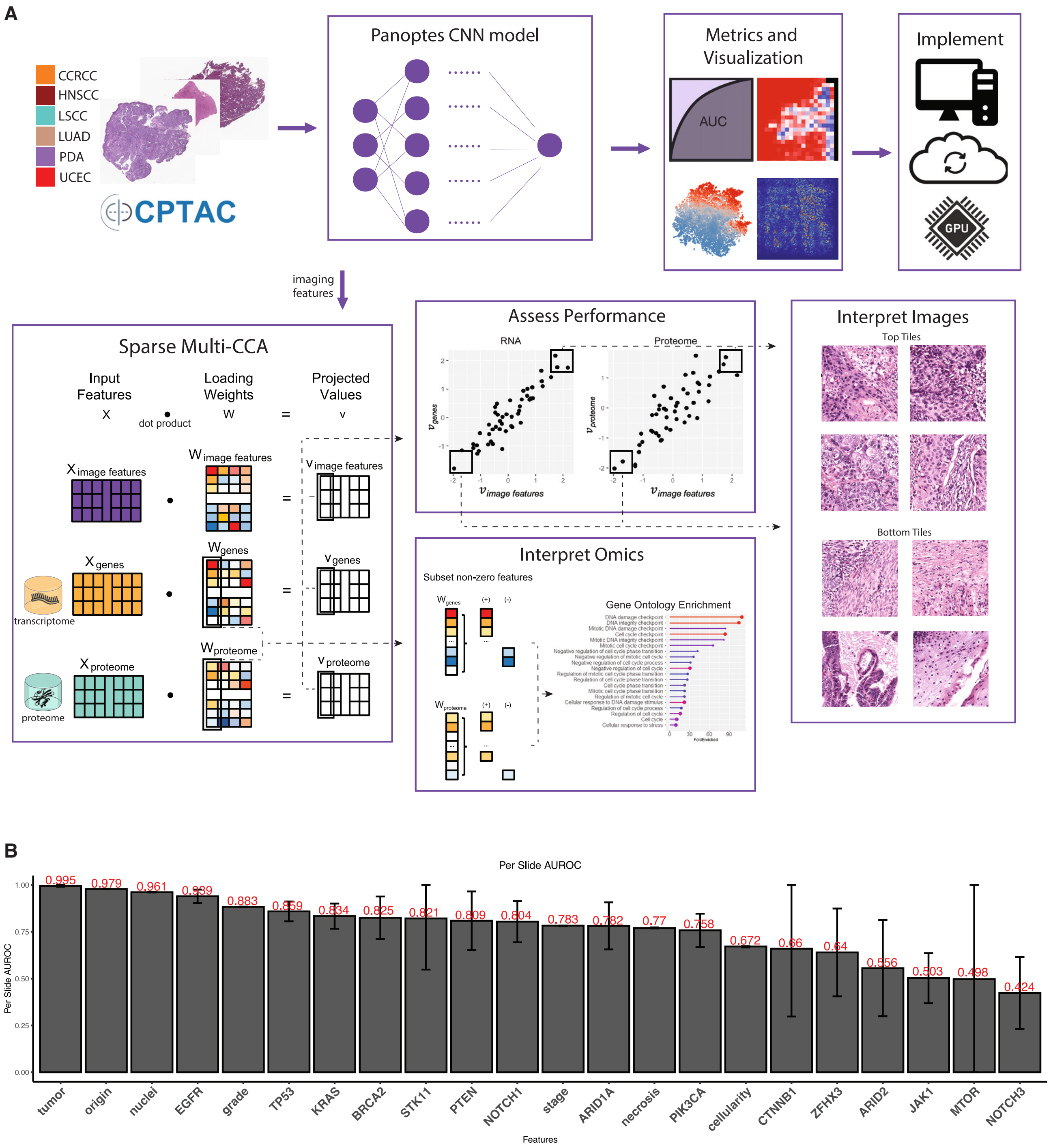

Figure 1. Workflow, data split, and model performance

(A) Overall workflow. Multi-resolution Panoptes models were trained on H&E slide images from six cancer types. Multi-CCA correlated proteomics, transcriptomics, and extracted imaging features from CNN models to reveal significant pathways and molecular signatures.

(B) Per-slide level AUROCs of imaging-based prediction tasks with 95% confidence intervals.

Insights into underlying biological mechanisms driving predictive morphology alterations were investigated through sparse CCA, which extracts subgroups of genes, proteins, and imaging features whose expression values maximally correlate with one another. Pathology review was then performed for high- and low-scoring cases for different canonical variants to assess the pathologic and biological relevance of model-derived features.

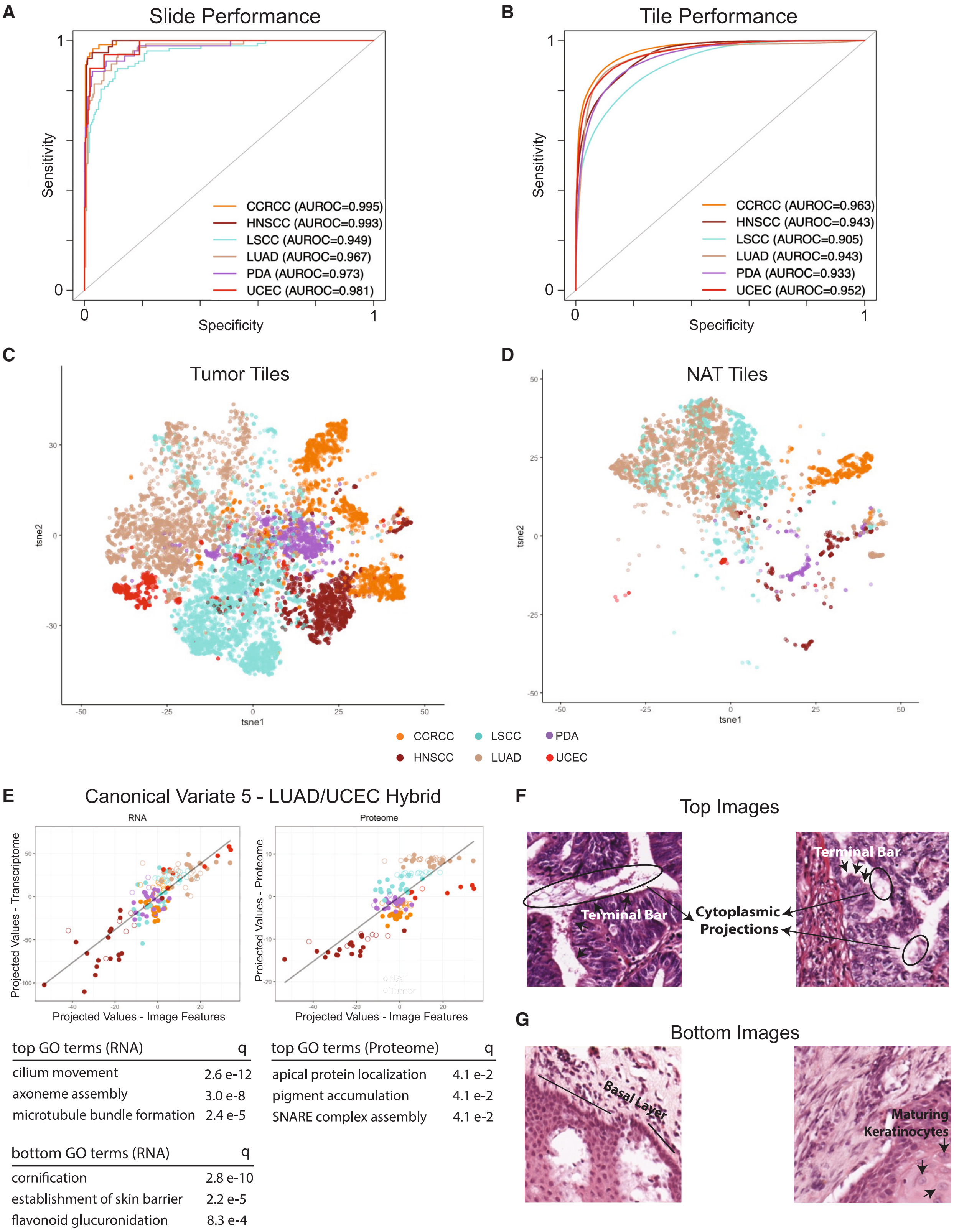

(legend on next page)

CellPress
OPEN ACCESS

Summarized comparison of model performance for all trained tasks is reported at the per-slide (Figure 1B) level.

Tissue-of-origin

Despite having molecularly targeted therapy for many malignancies, tumor lineage remains an important determinant for treatment selection, and prognostication based on tumor grade and stage. We trained models to predict tumor tissue-of-origin (lineage) and established a baseline for the global variation among cancers stemming from different organ systems. A model trained with combined NAT and tumor samples performs exceptionally well, with AUROCs ranging from 0.949 to 0.995 at the per-slide level (Figure 2A) and from 0.905 to 0.963 at per-tile level (Figure 2B). We visualized the tSNE separation of the learned latent features from the final convolutional layer to infer the extent of separation between different tissue types (Figures 2C and 2D). As expected, CCRCC, with its distinctive clear cell morphology, clusters separately, while samples from LSCC, LUAD, and HNSCC form a "rainbow"-like arc spanning the second tSNE component. PDA samples largely cluster together, but in the center of the two components. The low information captured by our imaging model may be due to sample heterogeneity within this cancer type. While not explicitly trained to do so, our models also discriminated between NAT and tumor images. NAT and tumor samples from LSCC were divided by LUAD samples, for which the latter clustered more tightly. Endometrial samples clustered the most distantly, with the UCEC tumor group dividing the two lung tumor groups along the first tSNE component. The normal endometrial samples clustered compactly but distinctly near pancreatic samples.

The tissue-of-origin model was validated on TCGA samples including both FFPE and fresh frozen section images (Figure S1D). The model generalized better on FFPE sections (AUROC: CCRCC 0.99, HNSCC 0.96, LSCC 0.85, LUAD 0.88, and PDA 0.97) than on fresh frozen tissues (AUROC: CCRCC 0.89, HNSCC 0.84, LSCC 0.73, LUAD 0.76, and PDA 0.92). This is consistent with known artifactual degradation of histologic features by frozen section sample preparation. tSNE clustering of latent output from TCGA samples recapitulates our CPTAC results; LUAD and LSCC cluster closely together, HNSCC samples reside near LSCC, and UCEC samples cluster near LUAD samples (Figure S2A). Taken together, external TCGA validation suggests that the tissue-of-origin model has learned generalizable morphologies that separate samples originating from different organ systems.

To investigate transcriptomic and the more directly tissue-relevant proteomic mechanisms driving predictive morphology alterations, we applied sparse CCA, a statistical method to extract high-dimensional rotations in the gene/protein/image space (canonical variates) whose projected values maximally correlate with one another.[18] When applied to pairs of high-dimensional data, CCA can project each dataset into a ranked series of paired new spaces, in which the projected values maximally correlate. Signals in histopathology images associated with molecular features can thus be identified despite potentially confounding complexity. For each canonical variate, the loading weights represent a directional rotation. Projected values, representing the coordinate location of each input along that particular direction are calculated with the dot product of the load weights and input matrices. Samples whose input features strongly associate with the loading weights will have more extreme projected values. This process is iterated to identify multiple canonical variates. For each canonical variate, genes and proteins with non-zero loading weights (responsible for that variate's directional rotation) are interpreted with gene ontology (GO) term enrichment. Our pathology team then manually annotated enriched tissue patterns present in the images with the largest and smallest projected values to discern histomorphologic correlations.

In a plot of canonical variate (no. 1) with the strongest correlation among all three data modalities (image features, RNA, and proteome), CCRCC tumor and non-neoplastic (NAT) samples separated from all others. GO term enrichment for fatty acid oxidation and amino acid catabolic processes were consistently found at both the transcriptomic and proteomic levels (Figure S2B), consistent with the increased role of fatty acid and amino acid catabolism in both normal kidney and CCRCC relative to other tissues in this study. HNSCC were found at the lowest portion of this canonical variant; GO terms enriched for low projected values include chromosomal segregation and cell-cycle DNA replication, cornification, and hair follicular development. A separate canonical variate (no. 5) reiterated the clustering of UCEC with LSCC and LUAD, grouping samples from these in the variate's highest projected values (Figure 2E). Protein data confirmed transcriptomic findings that these samples were enriched for cilium movement and microtubule bundle formation. Tiles with the highest projected values were enriched for cells with cytoplasmic projections and terminal bars, which were uniformly present in both endometrial and lung samples (Figure 2F), and reflect biological processes unique to mucus-secreting tissue. Bronchial cilia provide mucociliary clearance in the lung to maintain a healthy epithelium, and endometrial cilia help provide a suitable environment for embryonic development in the uterus. Conversely, HNSCC is enriched in the bottom portion of this canonical variate. Processes responsible for keratinization, establishment of skin barrier, and flavonoid glucuronidation can be extracted only at the transcriptomic level. These

Figure 2. Tissue-of-origin model performance and omics-integration

(A) AUROC for each cancer type at per-slide level.

(B) AUROC at per-tile level.

(C) Features extracted from penultimate layer are separated with tSNE; each dot represents a tumor tile colored by tissue origin.

(D) Feature extraction where each dot represents NAT tiles colored by tissue origin.

(E) CCA canonical variate highlighting similarities between UCEC and LUAD samples. Line graphs represent standardized coefficients for subsets of imaging, gene, and proteome features. Each dot represents an image-proteogenomic paired sample. GO term enrichment assessed on subset of genes and proteome features with non-zero values in loading matrix.

(F and G) Top and bottom images represent tiles with highest and lowest scores, respectively. Histopathology annotations reflect enriched GO terms.

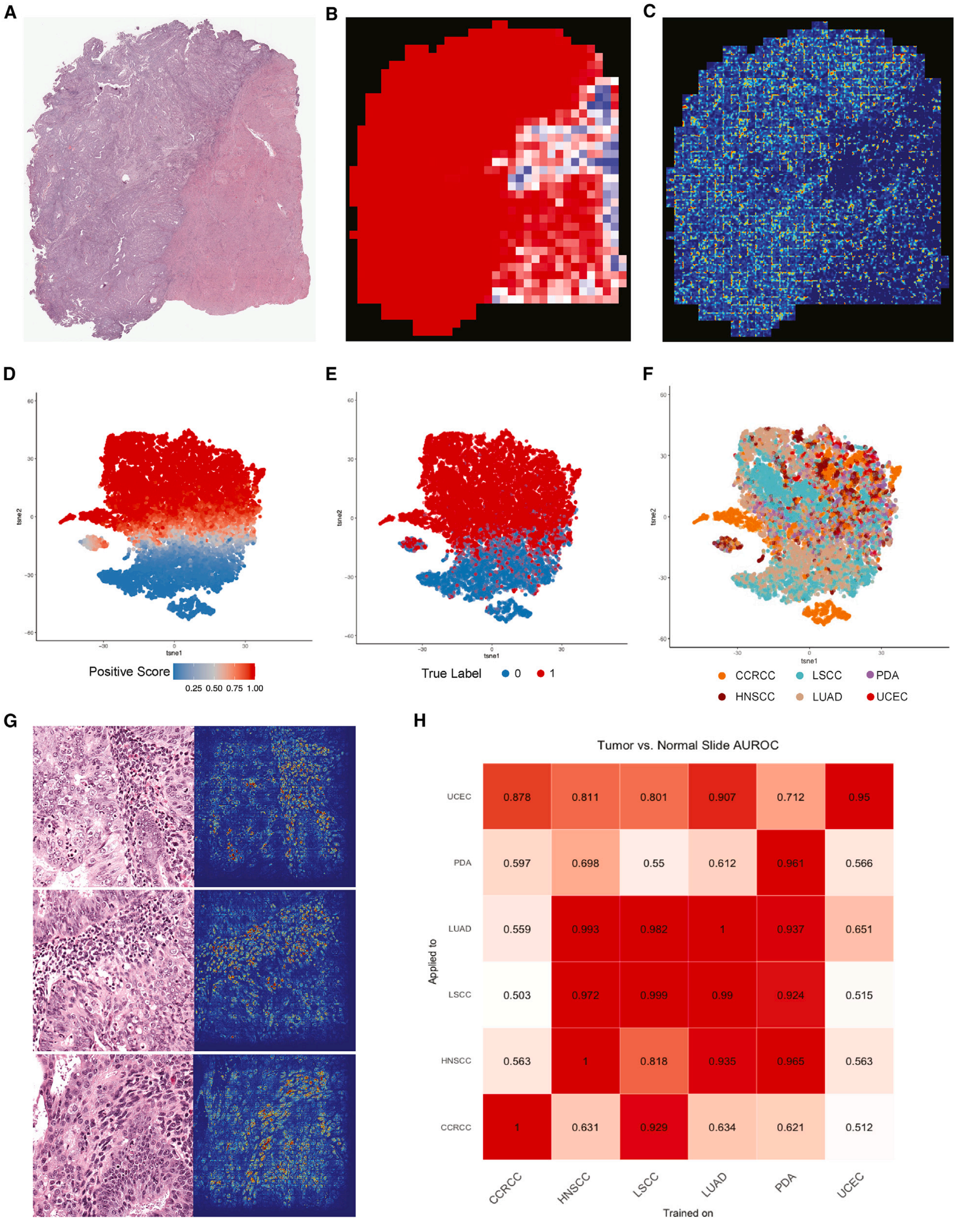

(legend on next page)

pathways match the physiology inherent to squamous cells comprising HNSCC. As expected, correlated tiles predominantly displayed extensive keratinization (Figure 2G), well beyond that noted in LSCC. Finally, PDA samples dominated the upper portion in canonical variate no. 7, with enrichment for collagen metabolism, chondrocyte development, and extracellular matrix disassembly at the transcriptomic level, and epithelial cell apoptotic processes and wound response at the proteomic level (Figure S2C). Visualization confirms regions comprising sparse malignant infiltrates (small, irregular nests of tumor cells) within desmoplastic (fibroblastic) stroma with abundant collagenous and myxoid extracellular matrix similar to what is seen in wound healing (scar formation), and other regions of normal pancreatic exocrine lobular and ductal tissue, involved in the secretion and transport of digestive enzymes (Figure S2D).

Tumorigenesis

We next assessed how CNN models of histologic features differentiate tumors from NAT (another class 1 task) and compared the model's decision-making process with standard pathologic examination. We trained a single pan-cancer imaging model to identify conserved architectures differentiating tumors from NAT samples. We then trained individual models for each cancer type to isolate tumorigenic signatures unique to individual organ systems. The pan-cancer model achieved AUROC of 0.995 (95% CI, 0.990–1) at the per-slide level and 0.972 (95% CI, 0.971–0.973) at per-tile level in classifying tumor and NAT (Figure 1B). On the external TCGA test set, AUROC was 0.94 (Figure S1D).

To identify histologic features heavily weighted in the model, we evaluated the spatial distribution of the prediction by the model at the per-slide level, and aggregated the per-tile level prediction scores and mapped them back to the original slide dimensions in the form of heatmaps (an example is shown in Figures 3A and 3B). The prediction was accurate with close to 1.00 prediction probability for all tumor tiles. Within each slide, we adopted class activation mapping (CAM) to demonstrate the attention of the deep learning model, and observed that when tile-level CAM was aggregated at the per-slide level, the model generally gave more attention to tumor regions than to normal areas (Figure 3C).

We then extracted the activation maps of the test set samples at the penultimate layer. Dimensional reductions were performed to display two-dimensional tSNE plots. We observed that tumor samples clustered on the top while the normal samples clustered at the bottom (Figure 3D), and that predicted and true labels correlated well (Figure 3E). There were no obvious clusters by tissue-of-origin except two small clusters relating to CCRCC samples (Figures 3F and S3A). Upon review of H&E tiles corresponding to dots on the tSNE plot, we confirmed that tumor tissue clusters accurately captured common tumorigenic features, while NAT areas showed large regions composed mainly of well-differentiated tissue organization. The small isolated cluster of samples was identified as mostly artifacts or corrupted tiles (Figure S3A). Per-tile level saliency maps focused on nuclei and cellular density, especially regions with high tumor-infiltrating lymphocytes (Figure 3G), suggesting that high nuclei density and nuclear shape/size are major features used by the model to distinguish tumor tissue from NAT.

Individual models trained on each of the six cancer types separately achieved per-slide level AUROC ranging from 0.95 (UCEC) to 1 (CCRCC, HNSCC, and LUAD). To determine if individual models used similar features or had utility outside of their site of origin, we applied each model to test sets of non-trained tissues (Figures 3H and S3B). The LUAD model had a per-slide AUROC of 0.907 when applied to UCEC samples, likely due to their histologic similarity. LUAD, LSCC, and HNSCC models generally transferred to one another with per-slide AUROC usually exceeding 0.9, which is also in line with our observation from tissue-of-origin models. Surprisingly, the LSCC model performed well on CCRCC samples (AUROC = 0.929), but our expert pathology review did not note distinctly generalizable features that could be articulated between the two cancer types, suggesting that this solution may represent a class 2b problem. The PDA model also performed well on LSCC, LUAD, and HNSCC, which may be explained by the morphology similarities between large pink cells and squamous tissue. Overall, our deep learning models appear to have captured tumor/NAT morphological differences that generalized across different cancer types to reveal morphological similarities in distinct tissue types.

Effective cancer therapy requires identification of pathway dependencies distinguishing tumor and normal tissues that can then be selectively targeted to affect tumor cell death. We therefore evaluated our pan-cancer model for molecular features distinguishing tumors from NAT. In the first canonical variate, the plots of projected values between imaging and transcriptomic or proteomic features show clear delineations between normal and tumor samples (Figure 4A). Evaluation of top transcriptomic and proteomic pathways and top tiles identified subsets of genes and proteins enriched for cell-cycle DNA replication and double-strand break repair (Figures 4B and 4C). Here, translational evidence validated transcriptomic findings. Manual review of the tiles with the highest projected values revealed tumor regions with scattered mitotic figures and pyknotic nuclei, and confirm that the imaging model has learned a subset of features correlating with these tumorigenesis pathways.

Figure 3. Feature visualization and cross-testing of tumorigenesis models
(A) Example UCEC slide with tumor tissue on left and normal tissue on right.
(B) Prediction heatmap of example slide with hotter areas (red) highlighting tiles more likely to be tumor tissue.
(C) CAM of example slide by tiles with hotter areas emphasizing the tumor tissue.
(D–F) Feature extraction from tumorigenesis imaging model by tSNE; each dot represents a tile colored by prediction score, true label, and cancer type, respectively.
(G) Example tiles of integrated saliency results highlighting accumulation of nuclei, with densest regions largely composed of stromal lymphoplasmacytic infiltrates.
(H) Heatmap showing per-slide AUROCs of applying single cancer type trained models to the other cancer types.

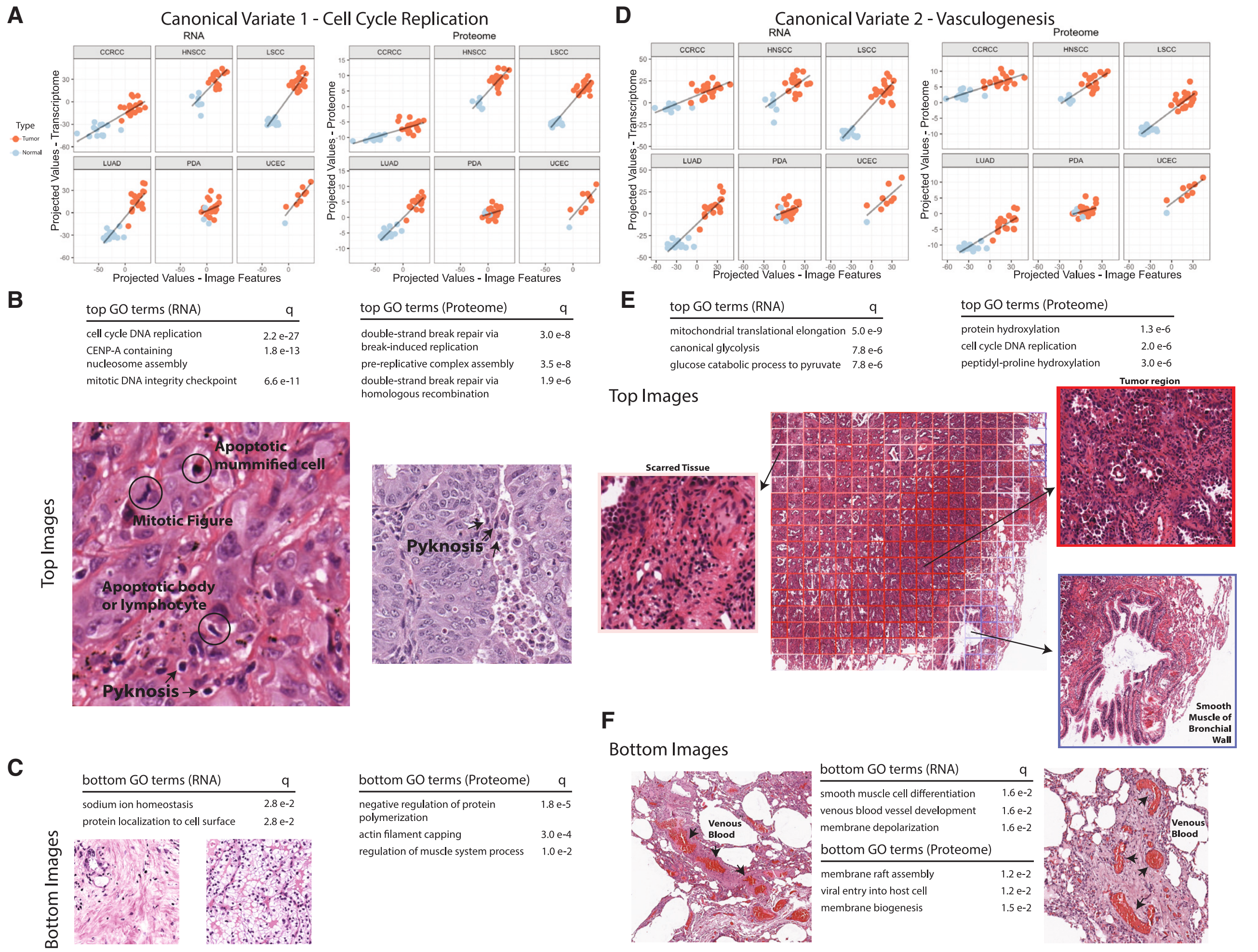

Figure 4. Major canonical variates associated with tumorigenesis

(A) Canonical variate with strongest correlation separating NAT/tumor samples across all six cancer types.

(B) Tiles from highest-scoring regions show mitotic morphologies consistent with enriched transcriptomic and proteomic enrichment.

(C) Tiles from lowest-scoring region.

(D) Second canonical variate distinguishing NAT/tumor samples.

(E and F) Tile scoring parallels enriched biological processes. Tile borders indicate scores; top-scoring regions (red) match tumorigenic areas with increased glycolytic activity, and bottom-scoring (blue) areas correspond with smooth muscle and blood vessel architectures.

Another canonical variate (no. 2) showed pronounced separation of LSCC and LUAD from NAT, and moderate separation of HNSCC tumors from NAT (Figures 4D and 4E). Within these cancer types, negative transcriptomic and proteomic features correlated with venous blood vessel development and smooth muscle cell differentiation (blue tiles), while positive features correlated with canonical glycolysis (red tiles). Upon visualization, blue regions visibly isolate regions with discrete venous blood (Figure 4F). When zoomed out, we observe the contrast where red regions correlating with increased metabolic activity highlight tiles with tumor cells, blue regions segment out areas of normal lung with smooth muscle tissue, and middle white tiles correlate with fibrotic or desmoplastic regions (Figure 4E). The imaging model has learned features that combine to represent interpretable biological signals and correspond to recognizable H&E features, and are not the results of random noise.

Clinical and histopathological features

Grade and stage are key clinical prognostic indices, and may correlate with underlying molecular changes driving aggressive tumor behavior. We trained models to predict pathologic grade (a class 1 task) and stage (a class 2 task) (Figure 5A). For prediction of grade, the best per-slide AUROC was 0.883 (95% CI, 0.882–0.84) and the best per-tile AUROC was 0.799 (95% CI, 0.799–0.800). For stage, the best per-slide AUROC was 0.783 (95% CI, 0.779–0.783) and the best per-tile AUROC was 0.727 (95% CI, 0.727–0.727). TCGA grade data were not available to test generalizability. The stage model was not generalizable to the external TCGA stage data (Figure S1D). This may be due in

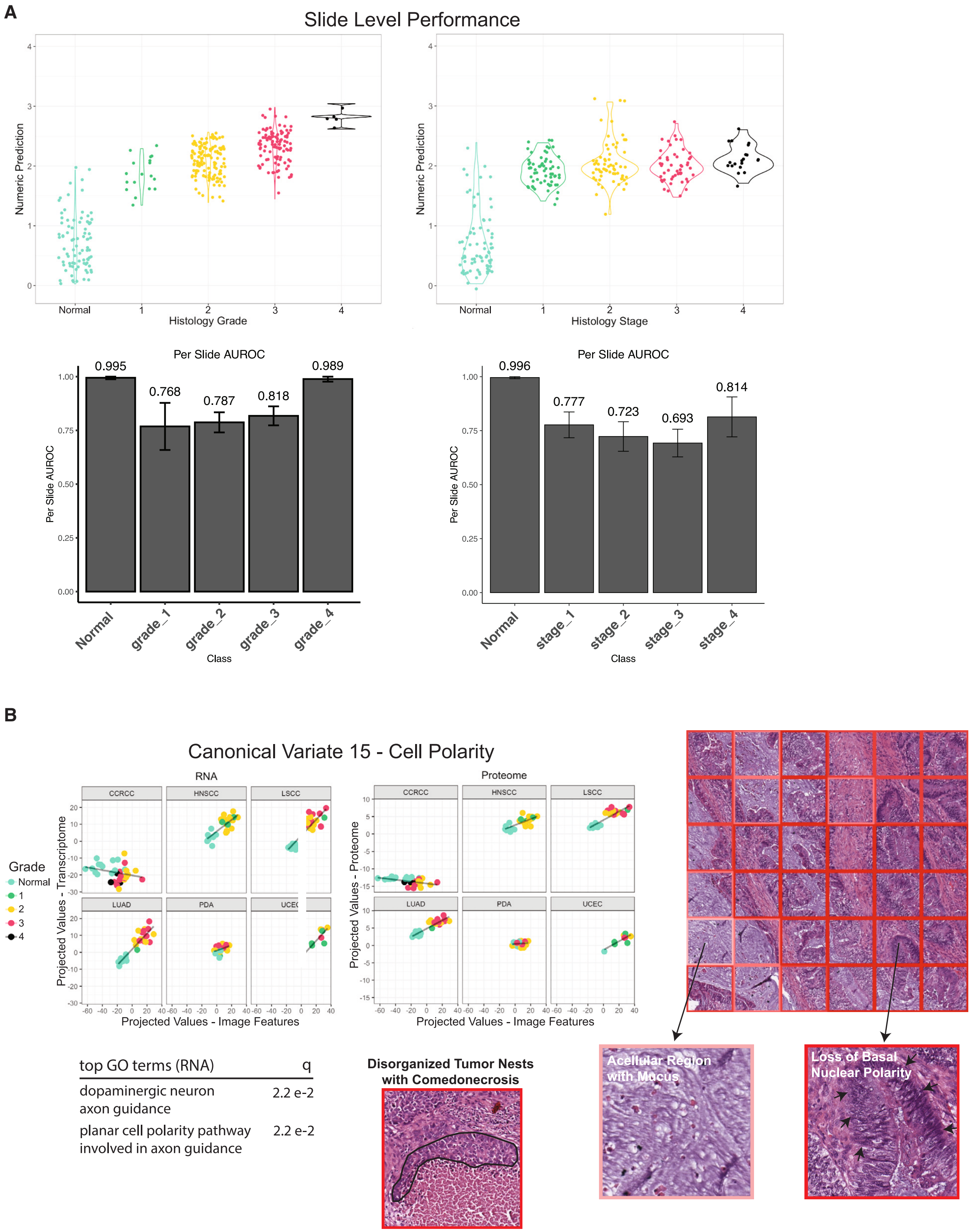

(legend on next page)

part to heterogeneity in the use of the AJCC 7th or 8th edition staging systems between TCGA and CPTAC data collection.

Given that tumor stage consists of information about the primary tumor, regional nodal metastasis, and distant disease (TNM criteria describing disease distribution), we queried if the model represented a human-interpretable task (class 2a) on the basis of available clinicopathologic data or image features. We found that grade and stage were not correlated (Figure S4A). Cross-evaluation of the stage model to predict grade, and also the grade model to predict stage, showed that these do not generalize (Figure S4B). We then performed blinded pathology review (E.G.D. and A.J.L.) on eight to nine WSIs of predicted stage 0 (NAT), 1, 2, and 3 cases, and four stage 4 cases to subjectively evaluate architectural and cytologic features (Figure S4C). While there were few pathologic clues to distinguish stage 1 from 2, stage 3 tumors showed a more microscopically aggressive appearance. Whereas stage 1 and 2 tumors tended to show large, cohesive rounded nested or glandular tumor architecture with a circumscribed pushing boundary with normal tissues, and sharp, well-defined basal laminae, stage 3 tumors were more likely to comprise small, irregular nests, cords, or single cells, which more diffusely infiltrated surrounding stroma. The basal aspect of stage 3 tumor nests showed a feathery or jagged appearance where malignant cells budded off and invaded adjacent stroma. Stage 4 was similar, with angulated tumor nests and single-cell infiltration noted in the majority of cases. Although these features are components of high-grade tumors, they are not the sole feature contributing to grade, which may explain why grade and stage did not correlate. Lymphatic or vascular invasion was not identified in the sections reviewed. Our exploratory findings define this model as a class 2A task—one in which the model was able to perform an unexpected task not normally performed by humans, yet yielded divisions with biologically relevant, human-interpretable differences that might prove interesting for further study.

We conducted sparse CCA to identify biologic correlates. The strongest signals were observed between chromosomal segregation and meiotic signaling with higher grade and stage at both the transcriptomic and proteomic levels. Linear separation was significantly present within five of six cancer types. PDA was the outlier without correlation, possibly due to the lower tumor purity of PDA specimens, which may dilute malignant proteogenomic signatures. Tiles with highest projected values showed densely packed apoptotic bodies and necrosis, while tiles with bottom scores showed well-differentiated tissue architecture. To distinguish characteristics responsible for local tumor growth and metastasis, we searched for canonical variates with signatures uniquely present in either grade or stage analyses. A canonical variate (no. 15) enriched for planar cell polarity was identified among high-grade HNSCC, LSCC, LUAD, and UCEC samples. Dysregulation of planar cell polarity is known to be associated with increased cell migration and proliferation, characteristic features of high-grade tumors. The canonical variate selected only three genes (*VANGL2*, *WNT5A*, and *RYK*) with non-zero weights, and could only be associated with a subset of imaging features at the transcriptomic level. Visualization of regions with highest projected values (Figure 5B) captured regions of disorganized tumor cells without lumen formation and with extensive necrosis, and areas where malignant glands showed loss of basal nuclear polarity with nuclei appearing pseudostratified across adjacent cells instead of neatly aligned near the basement membrane, consistent with dysregulation of polarity. Tiles with lowest projected values were dominated by NAT samples, with normal non-neoplastic morphologies or cells with apparent polarity.

Biomarkers

H&E slides are effective for visualizing morphological features. Additional molecular information can be acquired by immunohistochemistry (IHC) staining or nucleotide sequencing. However, the utility of IHC is limited by the requirement for antibodies both sensitive and specific to each marker of interest. Apart from limited panels targeting frequent cancer mutations, sequencing is often cost-prohibitive and time-consuming, limiting clinical accessibility. A tool to infer molecular signatures directly from H&E-stained histopathology images would harness the power of omics research to more accessible diagnostic pathology images, augment traditional diagnostic pathology techniques, and expand retrospective molecular analysis via inference of biospecimens lacking genomic sequencing. Such tools could be used in clinical practice to predict existence not only of specific mutations but also of targetable pathway dependencies at the protein level. As proof-of-concept, we tested the capacity of our model to accurately predict common genomic biomarkers directly from histopathology images, and then evaluated if these predictions represented class 2a or 2b tasks.

Promising results were obtained for prediction of specific mutations, including *EGFR*, *TP53*, *KRAS*, *STK11*, and *PTEN* (per-slide AUROC = 0.939, 0.859, 0.834, 0.821, 0.809, respectively) (Figure 1B). All mutation prediction tasks showed significant differences, except *BRCA2*, *JAK1*, and *MTOR*, suggesting that these models could be used for distinguishing tumors harboring common mutations (Figure 6A).

tSNE dimensional reduction was then conducted for mutation prediction tasks. Tiles with high-grade tumor features were observed in the predicted *TP53* mutant cluster. The upper left subcluster contained mostly small cells with spindled (sarcomatoid) morphology. The upper right subcluster depicted mostly small cells with hyperchromatic nuclei and regions of necrosis. A small subcluster with gland formation was found on the middle left (Figure 6B). In the predicted *KRAS* mutant cluster, larger nuclei, open chromatin, and glandular features were observed. However, tiles were not as high grade and densely cellular as the predicted *TP53* mutant tiles. Infiltration of single cells and

Figure 5. Model performance and multi-omics assessment of grade and stage

(A) Per-slide performance of models trained on tumor grade and disease stage. Numeric predictions represent expected value from softmax layer ($\sum_{x=0}^{4} p(x)(x)$) where *x* represents grade or stage outcome). AUROC for each outcome denoted.

(B) CCA canonical variate uniquely observed in grade analysis. Tiles with highest projected values (shown by more intense red borders) reflect regions with disorganized tumor nests lacking lumen formation and glandular regions with loss of basal nuclear polarity. Paler tile borders reflect lower projected values.

CellPress
OPEN ACCESS

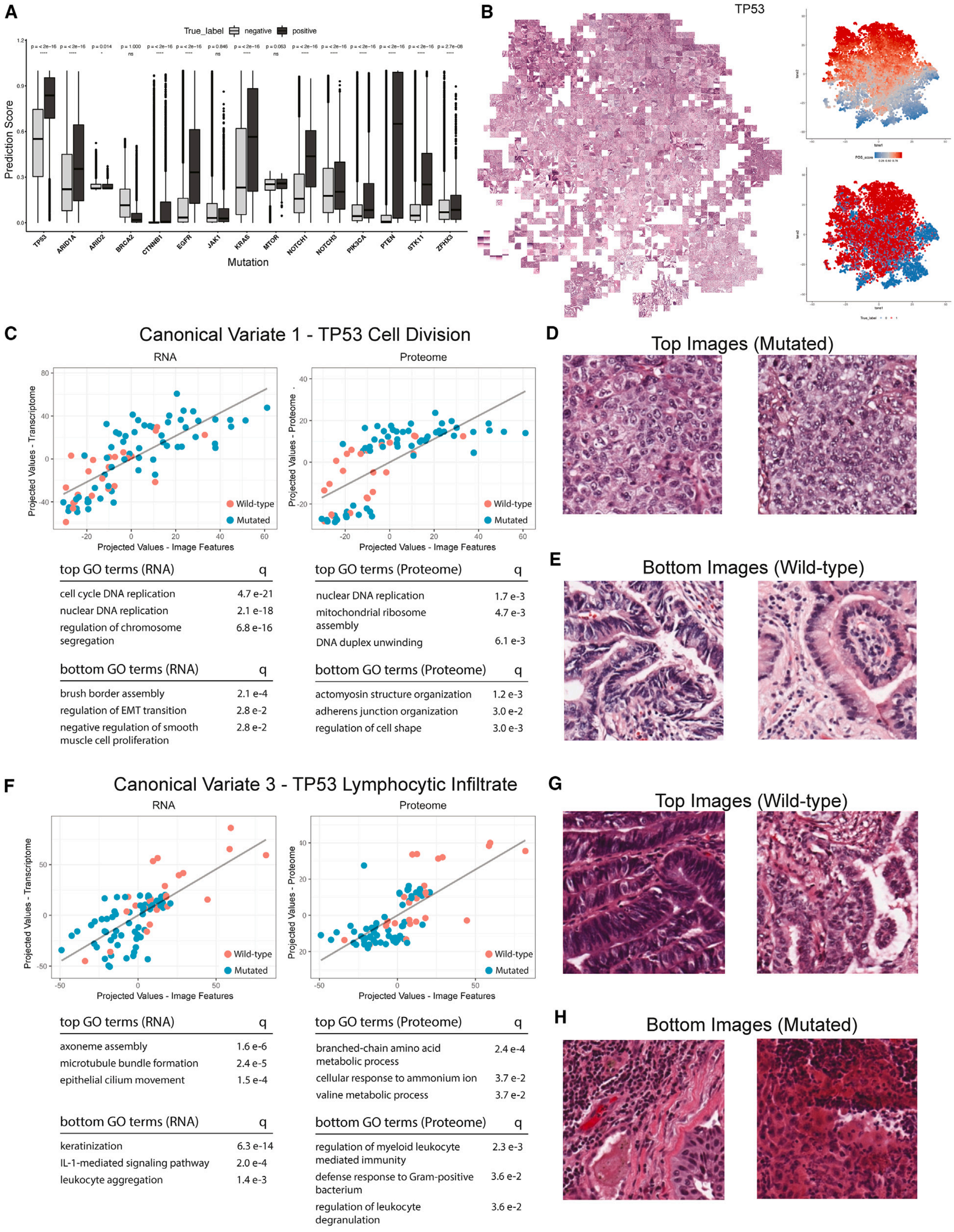

(legend on next page)

glands as well as mucin deposition and pooling were observed, which likely belonged to lung and GI tract tissues. A false-positive subcluster on the middle right consisted of adenosquamous carcinomas. Abundant neutrophils, large cells, and glandular architecture were found in predicted *STK11* mutants, and malignant cells showed clumped chromatin; these tiles were less cellular, more inflamed, and more necrotic compared with predicted *TP53* mutants. Similar patterns were observed in the *EGFR* mutant cluster. The predicted *PTEN* mutant cluster showed elongated dense glands, typical of endometrial carcinoma.

Validation on TCGA FFPE (Figure S1D) confirmed generalizability of *TP53* and *PTEN* (AUROC on CPTAC: 0.86, 0.81 and TCGA: 0.77, 0.80, respectively), although only *PTEN* generalized when images of frozen tissue were utilized (AUROC = 0.84). *KRAS*, *EGFR*, and *STK11* models did not generalize.

To determine if models were based on tumor-type independent, pathologically identifiable, and biologically relevant information, blinded pathology review was performed on subsets of CPTAC and TCGA cases with and without *TP53*, *PTEN*, or *STK11* mutations. Within both CPTAC (Figure S5A) and TCGA (Figure S5B) cases, we observed that, regardless of tissue-of-origin, *TP53* mutated samples were densely cellular, and frequently showed dense lymphocytic infiltrates within tumor stroma, as well as high mitotic rate, increased nuclear/cytoplasmic (N/C) ratio and, in many cases, scattered "monster cells" with enlarged, hyperchromatic, multilobulated nuclei that were much larger than the majority of malignant cells. In contrast, *TP53* wild-type samples typically consisted of malignant cells with lower N/C ratios, smaller, more uniform nuclei, and moderate-to-abundant cytoplasm. We therefore defined *TP53* mutation calling as potentially a class 2a task that pathologists may be able to learn to perform.

Review of cases predicted as *PTEN* mutated consistently showed tissue-specific histology in both CPTAC and TCGA images. Sections were characterized by complex villoglandular and tubulo-papillary structures with gaping lumens free of secretions or mucus—all features specific to endometrioid adenocarcinoma from the included cancers. We concluded that the PTEN model identified endometrioid adenocarcinoma, where PTEN mutations are very common (79% in CPTAC, 62% in TCGA), as a surrogate for mutation status, and was not tissue independent. Pathologic correlates were not identified to discriminate between *STK11* wild-type and mutated tumors.

Subsequently, correlation between proteogenomic signatures and imaging features was only explored for TP53. Multi-modal integration revealed relevant biological associations for mutated status. Because *TP53* mutation is uncommon in CCRCC, a separate model excluding CCRCC samples was trained for *TP53* mutation and used for multi-omics integration to improve the ability of sparse CCA to detect genuine biological signals correlating with *TP53* mutation. The strongest canonical variate (no. 1) separated mutated and wild-type tumor tiles by the degree of cell division and chromosomal segregation (Figure 6C), consistent with known cell-cycle dysregulation resulting from mutant *TP53* and the histologically visualized increased mitotic activity. Regions with highest projected values (Figure 6D) represented mutated samples. Histologic review revealed densely cellular, poorly differentiated areas. Conversely, regions with lowest projected values (Figure 6E) corresponded with wild-type samples, and were dominated by well-differentiated architecture and expression of proteins associated with maintenance of cell shape and cell-matrix interactions. We also extracted an inflammatory canonical variate (no. 3) that was enriched for IL-1-mediated signaling and other immune-related processes within mutated samples (Figure 6F). *TP53*-mutated samples clustered with low projected values and visualization highlighted areas with dense lymphoplasmacytic infiltration that was not observed in tiles within the highest projected values (Figures 6G and 6H). Taken together with the blinded whole-slide pathologic evaluation of TP53 wild-type vs. mutated samples, these findings show that our model consistently reproduces typical histologic features of *TP53* mutant cancers, specifically increased inflammation and mitotic activity, together with their proteomic and transcriptomic signatures.

For previous tasks, genes selected as correlated at RNA and proteome level generally shared similar GO enrichments, suggesting congruence at multiple levels of the central dogma. However, *TP53* canonical variate no. 3 (Figures 6F–6H) underscores the significance of proteomics as a complementary perspective into cellular processes that transcriptomics alone may overlook. Although both RNA and proteomics were enriched for inflammatory signaling in TP53 mutated tumors, the selected genes (Figure S6A) point to distinct cell types (lymphocytes vs. myeloid, respectively). To investigate further, we utilized results from BayesDeBulk to jointly model the transcriptomic and proteomic data and estimate cell fractions present in TP53 mutated and wild-type samples.[23] Our analysis revealed substantial increases in neutrophil and macrophage populations in TP53 mutated cases (Figure S6B), aligning with findings from proteome GO enrichment. These cell types correspond to tissue response from tumor necrosis, a prevalent characteristic in TP53 mutated tumors. Thus, proteomics provides a distinct vantage point into additional cellular pathways not discernible from

Figure 6. Performance, visualization, and feature extraction of biomarkers

(A) One-tail Wilcoxon tests on prediction scores between positively and negatively labeled samples at per-tile level with significance levels.

(B) Extraction and visualization of features learned by pan-cancer *TP53* mutation model with tSNE. Reference plots of prediction scores and true labels on the right.

(C) Canonical variate with strongest association between image and proteogenomic features.

(D) Top tiles demonstrate highly cellular disordered regions correlating with *TP53* mutated samples.

(E) Bottom tiles (wild-type) highlight organized and well-differentiated regions.

(F) Canonical variate correlating increased IL-1 activity with *TP53* mutated samples.

(G) Wild-type samples in canonical variate no. 3 highlight densely packed but relatively preserved tissue architectures.

(H) Conversely, mutated samples reside in the bottom portion and show areas of increased immune infiltrate activity.

CellPress
OPEN ACCESS

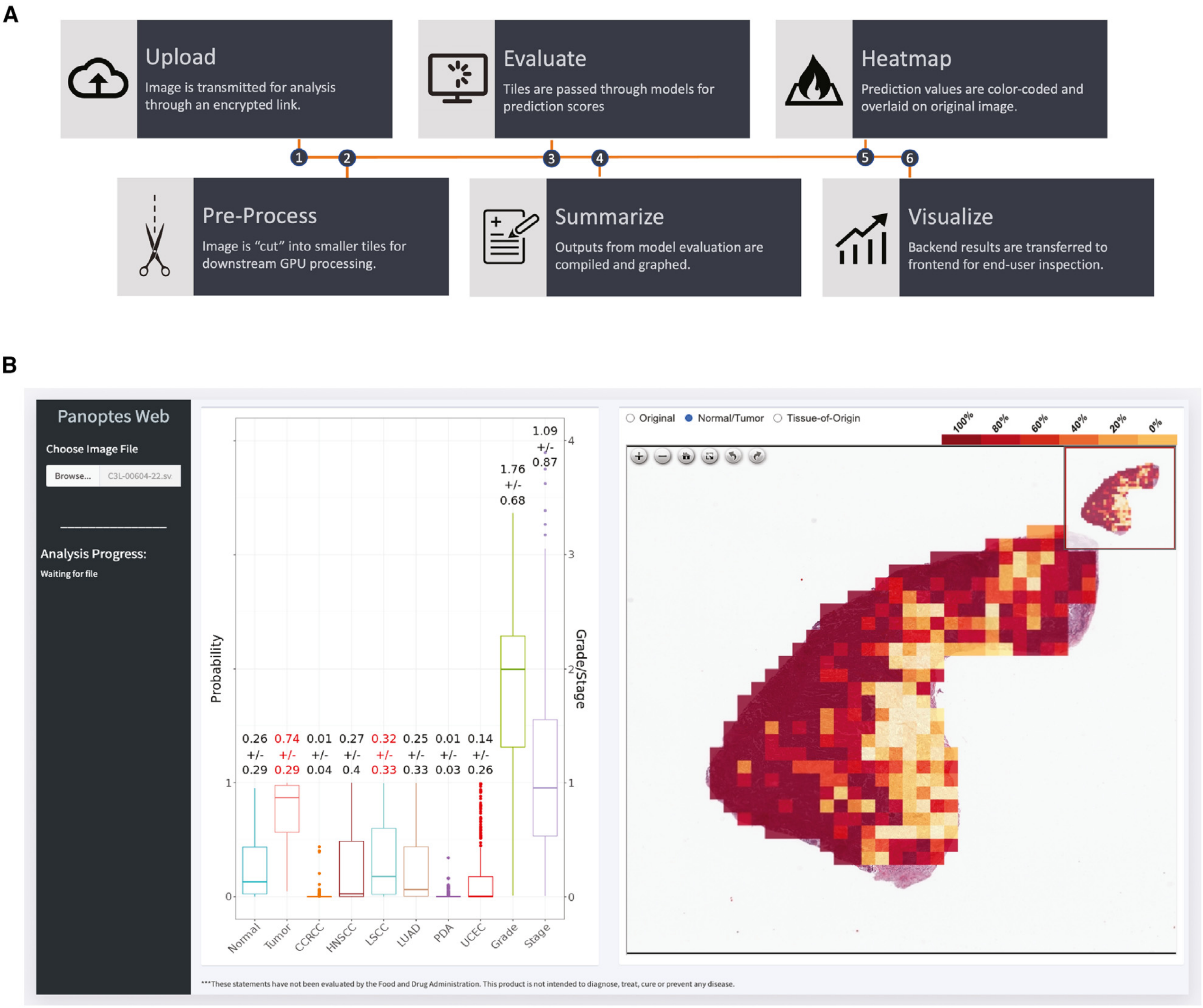

Figure 7. Panoptes Web
(A) App workflow.
(B) Boxplot assessment of probability scores and class outcomes, and individual tile probability visualization.

transcriptomics alone. In the case of TP53 mutated samples, this complementary information provided by proteomics offers a more comprehensive perspective on the immune cell types present and their potential contributions to the tumor microenvironment.

Panoptes Web

To facilitate integration into clinical and translational research workflows, we developed Panoptes Web, an intuitive visualization tool (http://panoptes.fenyo.cloud) for clinicians, scientists, and readers to assess our models' performances with standalone H&E images and visualize the predictions tile-by-tile using a simple workflow (Figure 7A). Results are plotted in an intuitive boxplot of probability scores and class outcomes along with a web-based viewer detailing tile-by-tile predictions (Figure 7B).

DISCUSSION

Previous efforts have utilized H&E histopathological images to predict clinical and genomic or transcriptomic outcomes in various cancer tissues, generally using one of two approaches. In a two-stage approach, image features were extracted by predefined rules using tools such as *CellProfiler*, and then submitted to machine learning classifiers that predict outcome.[24] Alternatively, CNNs were trained to infer sample labels directly from images.[25,26] Multi-resolutional models have been built to predict mutation burden in lung cancer.[27] Here, we built end-to-end, multi-resolution neural networks on pan-cancer data comprising six disease types to predict a wide range of histologic, clinical, and molecular outcomes. Importantly our modeling includes proteomics as well as genomics and transcriptomics. Proteomic features have been included as a

modality both validating and expanding upon gene expression profiling.

We sought to determine which complex models of large-scale multi-omics datasets could be rendered human-interpretable using human intuition in the absence of attention maps to enhance uptake of this technology in translational research and clinical practice. As part of our pathology-centric focus, we proposed a classification scheme for AI modeling tasks, based on the ability of human pathologists to produce similar results, either *a priori* (class 1), or after review of the model groupings (class 2). We performed exploratory blinded pathologic annotations of WSIs for cases that had been correctly assigned to mutation categories by our models to assess model generalizability and potential histologic correlates improving model explainability. While other groups have focused pathologic evaluation more exclusively to attention maps, we took an innovative approach, asking our expert pathologists to blindly evaluate AI-designated groups of CPTAC and TCGA cases and report on overall architectural and cytomorphologic features that they felt tied tumors in these groups together. These "gestalt" impressions were then used to generate a list of potential histologic features that pathologists could use to replicate the model classifier. This analysis led us to further subclassify class 2 tasks as those that might be amenable to pathologic intuition (class 2a) or those based on features that expert surgical pathologists cannot independently intuit (class 2b). While this approach requires formal validation on a larger scale, we believe that our initial results show promise as a framing concept for the application of proteogenomic modeling of histopathologic image data.

Latent image features extracted by the neural networks were correlated with proteogenomic data using multiple sparse CCA to directly correlate pathway level perturbations in protein expression with observed and interpreted pathologic images, and identify molecular signatures driving phenotype differences. Proteomics may yield better diagnostic and therapeutic insights compared with transcriptomics as it more directly tracks cellular states and responses.[28,29] Indeed, in most cases, our proteomic data complemented transcriptomic features. However, in some cases, such as the assessment of TP53 canonical variant no. 3, proteomics identified different immune drivers than was revealed by transcriptomic data alone. Incorporation of these pathway level features in tumor histopathologic modeling may allow clinical assessment of therapeutic cancer dependencies based on histologic features, including, for instance, metabolic pathways differentially regulated in tumors, which may be susceptible to targeted reprogramming.

Our models performed class 1 tasks (tumor vs. normal and tissue-of-origin) exceptionally well, comparable with human pathology review and models in the existing literature,[7,30] as expected in these proof-of-concept challenges. This technology could potentially be extended to non-trivial classification tasks such as identifying tissue-of-origin in metastatic carcinomas of unknown primary, for which proper therapy requires clarification of primary site. Similarly, while performance was somewhat degraded in frozen tissues, it is conceivable that the tumor vs. normal and tissue of origin classifiers could aid pathologists in performing rapid intraoperative consultations for margins or identification and classification of metastatic disease.

Compared with other publications, we have achieved superior performance at the pan-cancer level for some biomarker predictions such as *TP53* (AUROC = 0.86), which notably generalized to an external TCGA test set (AUROC = 0.77). We determined that *TP53* mutation calling, as well as pathologic stage prediction, might be class 2a human-reproducible tasks. Of note, while humans could identify features potentially discriminating *TP53* wild-type and mutant tumors, and stage 2 and 3 tumors, we were not able to evaluate if these were the same features weighted in the model; we plan in future to perform more detailed attention mapping from the penultimate layer of the models to better investigate the relation between pathology-identified features and model-weighted features. Further rigorous validation of the class 2a tasks discussed above, or identification of other clinically relevant molecular features that could be predicted from histomorphology and replicated as a class 2a task by human pathologists could result in the development of new human-performable pathology morphometrics to clinically aid in the prediction of tumor behavior or prognostic or therapeutic biomarkers in situations where molecular testing or access to slide scanning technology is not readily available, such as in developing countries.

Increasingly, data suggest that human experts working in conjunction with machine learning tools can in fact outperform both the human and machine separately. Notably, a team training computational system for automated detection of metastatic breast cancers found that the AUC performance of an independent human pathologist's diagnoses increased from 0.966 to 0.995 when guided by feedback from a trained model that otherwise performed at 0.925 alone.[31] Similarly, the sensitivity of our *TP53* models is exceptionally high at 0.96 (0.98 if excluding CCRCC), and could be conceivably used as a fast and inexpensive preliminary screen to guide clinical genomic testing. A two-tiered testing system, in which the first is highly sensitive and the second is highly specific, is routinely used in medicine. Indeed, we forecast that biomedical machine learning efforts will produce decision support tools that supplement the performance of a pathologist-centered diagnostic medical system.[32]

A unique and major strength of our study is the integration of expert pathology review together with machine learning to assess the relevance of our models. Our classification of computational tasks as they relate to clinical pathologists' intuitions provides a conceptual framework in which to utilize and understand computational pathology, and may discover insights into disease biology in the research setting, particularly when combined with omics data. As further proof of this concept, our visualized tSNE distribution of morphology features, overlaid with heatmap, CAM, and integrated-saliency maps, ensures that the models align with medical intuition. In addition, the application of multiple sparse CCA to associate morphologies with expression perturbations at the transcriptional and translational levels deepens our understanding of molecular mechanisms underpinning tumor biology and specific histologic features.

This depth of integration between machine learning and clinical expertise is rarely performed in existing published studies, leading to difficulties in relating findings to the clinic or to translational medical research. These efforts to confirm that recognizable and biologically relevant features are incorporated into the model's predictions are essential to gain the trust of physicians and

patients and overcome the primary hindrance to mass adoption of machine learning-based clinical tools in medicine and research. We also demonstrate that potentially novel histopathologic predictors may be revealed by human review of model groupings, which may propel additional research into underlying biologic pathways. Moreover, such methods may also be capable of discovering and revealing clinically relevant histopathologic features beyond those currently assessed in routine clinical practice. As an example of this, our supposition based on blinded pathology reviews was that our model predicting pathologic stage was potentially predicated on the presence of microscopic evidence of decreased tumor cell cohesion and increased invasiveness.

Finally, we have also developed a GUI interface for trained clinicians to explore our models with their own clinical images. A built-in slide viewer is included to allow for visual comparisons between our predictions and users' medical decisions.

Limitations of the study

In our study, we have identified several limitations that need to be acknowledged to provide a comprehensive interpretation of our findings. Due to resource limits, all histological annotations and molecular characterizations were obtained only at per-slide or per-patient levels. Therefore, the presented image models were not able to capture the possible intra-slide morphological heterogeneity, nor could they associate it with molecular variations due to subclones or heterogeneous tissue of origin within each sample. However, our results demonstrate the robustness of the underlying biological associations between morphology features and molecular expressions, as demonstrated by significant differences in correlations when the data are randomly permuted (Figure S6). Future endeavors incorporating spatial transcriptomics are necessary to directly associate individual imaging tiles with local molecular features, leading to a more refined and precise analysis.

The inclusion of benign samples adjacent to cancer tissues in our tissue-of-origin analysis may introduce some influence from the cancer microenvironment, potentially affecting the tissue-of-origin prediction models. Consequently, the application of these models to infer the biology of normal healthy tissues may not be reflected. While our "gestalt approach" for interpreting predictions has shown interesting insights, we also acknowledge the value of using interpretable machine learning methods to gain a deeper understanding of our models' inner workings. Moreover, we are aware of potential batch effects in publicly available datasets used for training and validation. Although our models demonstrate excellent generalization across datasets, suggesting that they can detect a non-random signal that surpasses the noise introduced by batch effects, any clinical application of these models will require rigorous further validation.

In conclusion, our study provides valuable insights into the complex relationships between histopathology and molecular features across various cancer types, laying a strong foundation for further understanding cancer mechanisms and supporting the development of personalized medicine.

CONSORTIA

The members of the National Cancer Institute Clinical Proteomic Tumor Analysis Consortium for Pan-Cancer are François Aguet, Yo Akiyama, Eunkyung An, Shankara Anand, Meenakshi Anurag, Özgün Babur, Jasmin Bavarva, Chet Birger, Michael J. Birrer, Anna Calinawan, Lewis C. Cantley, Song Cao, Steven A. Carr, Michele Ceccarelli, Daniel W. Chan, Arul M. Chinnaiyan, Hanbyul Cho, Shrabanti Chowdhury, Marcin P. Cieslik, Karl R. Clauser, Antonio Colaprico, Daniel Cui Zhou, Felipe da Veiga Leprevost, Corbin Day, Saravana M. Dhanasekaran, Li Ding, Marcin J. Domagalski, Yongchao Dou, Brian J. Druker, Nathan Edwards, Matthew J. Ellis, Myvizhi Esai Selvan, David Fenyö, Steven M. Foltz, Alicia Francis, Yifat Geffen, Gad Getz, Michael A. Gillette, Tania J. Gonzalez Robles, Sara J.C. Gosline, Zeynep H. Gümüş, David I. Heiman, Tara Hiltke, Runyu Hong, Galen Hostetter, Yingwei Hu, Chen Huang, Emily Huntsman, Antonio Iavarone, Eric J. Jaehnig, Scott D. Jewell, Jiayi Ji, Wen Jiang, Jared L. Johnson, Lizabeth Katsnelson, Karen A. Ketchum, Iga Kolodziejczak, Karsten Krug, Chandan Kumar-Sinha, Alexander J. Lazar, Jonathan T. Lei, Yize Li, Wen-Wei Liang, Yuxing Liao, Caleb M. Lindgren, Tao Liu, Wenke Liu, Weiping Ma, D.R. Mani, Fernanda Martins Rodrigues, Wilson McKerrow, Mehdi Mesri, Alexey I. Nesvizhskii, Chelsea J. Newton, Robert Oldroyd, Gilbert S. Omenn, Amanda G. Paulovich, Samuel H. Payne, Francesca Petralia, Pietro Pugliese, Boris Reva, Ana I. Robles, Karin D. Rodland, Henry Rodriguez, Kelly V. Ruggles, Dmitry Rykunov, Shankha Satpathy, Sara R. Savage, Eric E. Schadt, Michael Schnaubelt, Tobias Schraink, Stephan Schürer, Zhiao Shi, Richard D. Smith, Xiaoyu Song, Yizhe Song, Vasileios Stathias, Erik P. Storrs, Jimin Tan, Nadezhda V. Terekhanova, Ratna R. Thangudu, Mathangi Thiagarajan, Nicole Tignor, Joshua M. Wang, Liang-Bo Wang, Pei Wang, Ying Wang, Bo Wen, Maciej Wiznerowicz, Yige Wu, Matthew A. Wyczalkowski, Lijun Yao, Tomer M. Yaron, Xinpei Yi, Bing Zhang, Hui Zhang, Qing Zhang, Xu Zhang, and Zhen Zhang.

STAR★METHODS

Detailed methods are provided in the online version of this paper and include the following:

- KEY RESOURCES TABLE
- RESOURCE AVAILABILITY
  - Lead contact
  - Materials availability
  - Data and code availability
- EXPERIMENTAL MODEL AND SUBJECT DETAILS
- METHOD DETAILS
  - Image and data acquisition
  - H&E slide processing and sample preparation
  - Computational method of deep learning models
  - Feature extraction and visualization
  - Omics integration with sparse multiCCA
  - Minimizing effects of tissue of origin on pan-cancer mutation prediction

SUPPLEMENTAL INFORMATION

Supplemental information can be found online at https://doi.org/10.1016/j.xcrm.2023.101173.

ACKNOWLEDGMENTS

This work was supported by the National Cancer Institute (NCI) Clinical Proteomic Tumor Analysis Consortium (CPTAC) under award nos. U24CA210955, U24CA210985, U24CA210986, U24CA210954, U24CA210967, U24CA210972, U24CA210979, U24CA210993, U01CA214114, U01CA214116, and U01CA214125, and a contract from Leidos (S21-167). J.M.W. is supported by the NYU Medical Scientist Training Program (T32GM136573); he is also supported, in part, by an NYU Clinical and Translational Science Institute grant (TL1TR001447) from the National Center for Advancing Translational Sciences, National Institutes of Health, in addition to a fellowship (F30CA271622) from the National Cancer Institute of the National Institutes of Health. G.S.O. is supported by the National Institute of Environmental Health Sciences (P30ES017885). This project has also been funded in part with federal funds from the National Cancer Institute, National Institutes of Health, under contract no. 75N91019D00024, task order 75N91020F00029. The content of this publication does not necessarily reflect the views or policies of the Department of Health and Human Services, nor does mention of trade names, commercial products, or organizations imply endorsement by the US Government.

AUTHOR CONTRIBUTIONS

Study conception and design, J.M.W., R.H., W.L., and D.F.; performed experiment or data collection, Y.L., A.C., and C.P.T.A.C., computation and statistical analysis, J.M.W., R.H., J.T., N.R., T.S., and W.L.; data interpretation and biological analysis, J.M.W., R.H., E.G.D., R.L., A.L.M., M.A.G., A.T., K.V.R., L.D., A.I.R., D.R.M., K.D.R., A.J.L., W.L., and D.F.; writing – original drafts, J.M.W., R.H., and W.L.; writing – review & editing, J.M.W., R.H., E.G.D., J.T., R.L., A.L.M., N.R., T.S., M.A.G., G.S.O., E.A., H.R., A.T., K.V.R., L.D., A.I.R., D.R.M., K.D.R., A.J.L., W.L., and D.F.; supervision, A.J.L., W.L., and D.F.; administration, H.R., A.I.R., D.R.M., K.D.R., and D.F.

DECLARATION OF INTERESTS

The authors declare no competing interests.

Received: July 10, 2022
Revised: July 31, 2023
Accepted: August 4, 2023
Published: August 14, 2023

REFERENCES

1. Niazi, M.K.K., Parwani, A.V., and Gurcan, M.N. (2019). Digital pathology and artificial intelligence. Lancet Oncol. *20*, e253–e261. https://doi.org/10.1016/S1470-2045(19)30154-8.
2. Srinidhi, C.L., Ciga, O., and Martel, A.L. (2021). Deep neural network models for computational histopathology: A survey. Med. Image Anal. *67*, 101813. https://doi.org/10.1016/j.media.2020.101813.
3. Coudray, N., Ocampo, P.S., Sakellaropoulos, T., Narula, N., Snuderl, M., Fenyö, D., Moreira, A.L., Razavian, N., and Tsirigos, A. (2018). Classification and mutation prediction from non–small cell lung cancer histopathology images using deep learning. Nat. Med. *24*, 1559–1567. https://doi.org/10.1038/s41591-018-0177-5.
4. Hong, R., Liu, W., and Fenyö, D. (2021). Predicting and Visualizing STK11 Mutation in Lung Adenocarcinoma Histopathology Slides Using Deep Learning. BioMedInformatics *2*, 101–105. https://doi.org/10.3390/biomedinformatics2010006.
5. Hong, R., Liu, W., DeLair, D., Razavian, N., and Fenyö, D. (2021). Predicting endometrial cancer subtypes and molecular features from histopathology images using multi-resolution deep learning models. Cell Rep. Med. *2*, 100400. https://doi.org/10.1016/j.xcrm.2021.100400.
6. Sanchez-Vega, F., Mina, M., Armenia, J., Chatila, W.K., Luna, A., La, K.C., Dimitriadoy, S., Liu, D.L., Kantheti, H.S., Saghafinia, S., et al. (2018). Oncogenic Signaling Pathways in The Cancer Genome Atlas. Cell *173*, 321–337.e10. https://doi.org/10.1016/j.cell.2018.03.035.
7. Kather, J.N., Heij, L.R., Grabsch, H.I., Loeffler, C., Echle, A., Muti, H.S., Krause, J., Niehues, J.M., Sommer, K.A.J., Bankhead, P., et al. (2020). Pan-cancer image-based detection of clinically actionable genetic alterations. Nat. Can. (Ott.) *1*, 789–799. https://doi.org/10.1038/s43018-020-0087-6.
8. Schmauch, B., Romagnoni, A., Pronier, E., Saillard, C., Maillé, P., Calderaro, J., Kamoun, A., Sefta, M., Toldo, S., Zaslavskiy, M., et al. (2020). A deep learning model to predict RNA-Seq expression of tumours from whole slide images. Nat. Commun. *11*, 3877–3915. https://doi.org/10.1038/s41467-020-17678-4.
9. Chen, H., Gomez, C., Huang, C.M., and Unberath, M. (2022). Explainable medical imaging AI needs human-centered design: guidelines and evidence from a systematic review. NPJ Digit. Med. *5*, 156. https://doi.org/10.1038/S41746-022-00699-2.
10. Rodriguez, H., and Pennington, S.R. (2018). Revolutionizing Precision Oncology through Collaborative Proteogenomics and Data Sharing. Cell *173*, 535–539. https://doi.org/10.1016/j.cell.2018.04.008.
11. Clark, D.J., Dhanasekaran, S.M., Petralia, F., Pan, J., Song, X., Hu, Y., da Veiga Leprevost, F., Reva, B., Lih, T.S.M., Chang, H.Y., et al. (2019). Integrated Proteogenomic Characterization of Clear Cell Renal Cell Carcinoma. Cell *179*, 964–983.e31. https://doi.org/10.1016/j.cell.2019.10.007.
12. ICGC/TCGA Pan-Cancer Analysis of Whole Genomes Consortium; Getz, G., Korbel, J.O., Stuart, J.M., Jennings, J.L., Stein, L.D., Perry, M.D., Nahal-Bose, H.K., Ouellette, B.F.F., Li, C.H., et al. (2020). Pan-cancer analysis of whole genomes. Nature *578*, 82–93. https://doi.org/10.1038/s41586-020-1969-6.
13. Dou, Y., Kawaler, E.A., Cui Zhou, D., Gritsenko, M.A., Huang, C., Blumenberg, L., Karpova, A., Petyuk, V.A., Savage, S.R., Satpathy, S., et al. (2020). Proteogenomic Characterization of Endometrial Carcinoma. Cell *180*, 729–748.e26. https://doi.org/10.1016/j.cell.2020.01.026.
14. Gillette, M.A., Satpathy, S., Cao, S., Dhanasekaran, S.M., Vasaikar, S.V., Krug, K., Petralia, F., Li, Y., Liang, W.W., Reva, B., et al. (2020). Proteogenomic Characterization Reveals Therapeutic Vulnerabilities in Lung Adenocarcinoma. Cell *182*, 200–225.e35. https://doi.org/10.1016/j.cell.2020.06.013.
15. Satpathy, S., Krug, K., Jean Beltran, P.M., Savage, S.R., Petralia, F., Kumar-Sinha, C., Dou, Y., Reva, B., Kane, M.H., Avanessian, S.C., et al. (2021). A proteogenomic portrait of lung squamous cell carcinoma. Cell *184*, 4348–4371.e40. https://doi.org/10.1016/j.cell.2021.07.016.
16. Huang, C., Chen, L., Savage, S.R., Eguez, R.V., Dou, Y., Li, Y., da Veiga Leprevost, F., Jaehnig, E.J., Lei, J.T., Wen, B., et al. (2021). Proteogenomic insights into the biology and treatment of HPV-negative head and neck squamous cell carcinoma. Cancer Cell *39*, 361–379.e16. https://doi.org/10.1016/j.ccell.2020.12.007.
17. Cao, L., Huang, C., Cui Zhou, D., Hu, Y., Lih, T.M., Savage, S.R., Krug, K., Clark, D.J., Schnaubelt, M., Chen, L., et al. (2021). Proteogenomic characterization of pancreatic ductal adenocarcinoma. Cell *184*, 5031–5052.e26. https://doi.org/10.1016/j.cell.2021.08.023.
18. Zhuang, X., Yang, Z., and Cordes, D. (2020). A technical review of canonical correlation analysis for neuroscience applications. Hum. Brain Mapp. *41*, 3807–3833. https://doi.org/10.1002/hbm.25090.
19. Witten, D.M., Tibshirani, R., and Hastie, T. (2009). A penalized matrix decomposition, with applications to sparse principal components and canonical correlation analysis. Biostatistics *10*, 515–534. https://doi.org/10.1093/biostatistics/kxp008.
20. Ash, J.T., Darnell, G., Munro, D., and Engelhardt, B.E. (2021). Joint analysis of expression levels and histological images identifies genes associated with tissue morphology. Nat. Commun. *12*, 1609–1612. https://doi.org/10.1038/s41467-021-21727-x.
21. Subramanian, A., Narayan, R., Corsello, S.M., Peck, D.D., Natoli, T.E., Lu, X., Gould, J., Davis, J.F., Tubelli, A.A., Asiedu, J.K., et al. (2017). A Next

CellPress
OPEN ACCESS

Generation Connectivity Map: L1000 Platform and the First 1,000,000 Profiles. Cell *171*, 1437–1452.e17. https://doi.org/10.1016/j.cell.2017.10.049.

22. Li, Y., Dou, Y., Leprevost, F.D., Geffen, Y., Calinawan, A.P., Aguet, F., Akiyama, Y., Anand, S., Birger, C., Cao, S., et al. (2023). Proteogenomic data and resources for pan-cancer analysis. Cancer Cell *41*, 1397–1406.

23. Petralia, F., Krek, A., Calinawan, A.P., Charytonowicz, D., Sebra, R., Feng, S., Gosline, S., Pugliese, P., Paulovich, P.G., et al. (2023). BayesDeBulk: A flexible Bayesian algorithm for the deconvolution of bulk tumor data. bioRxiv. https://doi.org/10.1101/2021.06.25.449763.

24. Chen, L., Zeng, H., Zhang, M., Luo, Y., and Ma, X. (2021). Histopathological image and gene expression pattern analysis for predicting molecular features and prognosis of head and neck squamous cell carcinoma. Cancer Med. *10*, 4615–4628. https://doi.org/10.1002/CAM4.3965.

25. Azuaje, F., Kim, S.Y., Perez Hernandez, D., and Dittmar, G. (2019). Connecting histopathology imaging and proteomics in kidney cancer through machine learning. J. Clin. Med. *8*, 1535. https://doi.org/10.3390/jcm8101535.

26. Tabibu, S., Vinod, P.K., and Jawahar, C.V. (2019). Pan-Renal Cell Carcinoma classification and survival prediction from histopathology images using deep learning. Sci. Rep. *9*, 10509. https://doi.org/10.1038/S41598-019-46718-3.

27. Jain, M.S., and Massoud, T.F. (2020). Predicting tumour mutational burden from histopathological images using multiscale deep learning. Nat. Mach. Intell. *2*, 356–362. https://doi.org/10.1038/s42256-020-0190-5.

28. Cho, W.C.S. (2007). Proteomics Technologies and Challenges. Dev. Reprod. Biol. *5*, 77–85. https://doi.org/10.1016/S1672-0229(07)60018-7.

29. Dele-Oni, D.O., Christianson, K.E., Egri, S.B., Vaca Jacome, A.S., DeRuff, K.C., Mullahoo, J., Sharma, V., Davison, D., Ko, T., Bula, M., et al. (2021). Proteomic profiling dataset of chemical perturbations in multiple biological backgrounds. Sci. Data *8*, 226. https://doi.org/10.1038/S41597-021-01008-4.

30. Fu, Y., Jung, A.W., Torne, R.V., Gonzalez, S., Vöhringer, H., Shmatko, A., Yates, L.R., Jimenez-Linan, M., Moore, L., and Gerstung, M. (2020). Pancancer computational histopathology reveals mutations, tumor composition and prognosis. Nat. Can. *1*, 800–810. https://doi.org/10.1038/s43018-020-0085-8.

31. Wang, D., Khosla, A., Gargeya, R., Irshad, H., and Beck, A.H. (2016). Deep Learning for Identifying Metastatic Breast Cancer. Preprint at arXiv. https://doi.org/10.48550/arXiv.1606.05718.

32. Cui, M., and Zhang, D.Y. (2021). Artificial intelligence and computational pathology. Lab. Invest. *101*, 412–422. https://doi.org/10.1038/S41374-020-00514-0.

33. Clark, K., Vendt, B., Smith, K., Freymann, J., Kirby, J., Koppel, P., Moore, S., Phillips, S., Maffitt, D., Pringle, M., et al. (2013). The Cancer Imaging Archive (TCIA): maintaining and operating a public information repository. J. Digit. Imag. *26*, 1045–1057. https://doi.org/10.1007/S10278-013-9622-7.

34. Abadi, M., Barham, P., Chen, J., Chen, Z., Davis, A., Dean, J., Devin, M., Ghemawat, S., Irving, G., Isard, M., Kudlur, M., Levenberg, J., Monga, R., Moore, S., Murray, D.G., Steiner, B., Tucker, P., Vasudevan, V., Warden, P., Wicke, M., Yu, Y., and Zheng, X. (2016). TensorFlow: a system for large-scale machine learning. in Proceedings of the 12th USENIX conference on Operating Systems Design and Implementation (USENIX Association), pp. 265–283.

35. Szegedy, C., Vanhoucke, V., Ioffe, S., Shlens, J., and Wojna, Z. (2015). Rethinking the Inception Architecture for Computer Vision. Proc. IEEE Comput. Soc. Conf. Comput. Vis. Pattern Recognit. *2016-December*, 2818–2826. https://doi.org/10.1109/CVPR.2016.308.

36. Szegedy, C., Ioffe, S., Vanhoucke, V., and Alemi, A.A. (2016). Inception-v4, Inception-ResNet and the Impact of Residual Connections on Learning. 31st AAAI Conf. Artif. Intell. AAAI 2017, 4278–4284. https://doi.org/10.1609/aaai.v31i1.11231.

37. Chollet, F., and others (2015). Keras. Github. https://github.com/fchollet/keras.

38. Vahadane, A., Peng, T., Sethi, A., Albarqouni, S., Wang, L., Baust, M., Steiger, K., Schlitter, A.M., Esposito, I., and Navab, N. (2016). Structure-Preserving Color Normalization and Sparse Stain Separation for Histological Images. IEEE Trans. Med. Imag. *35*, 1962–1971. https://doi.org/10.1109/TMI.2016.2529665.

39. Zhou, B., Khosla, A., Lapedriza, A., Oliva, A., and Torralba, A. (2016). Learning Deep Features for Discriminative Localization. In 2016 IEEE Conference on Computer Vision and Pattern Recognition (CVPR) (IEEE), pp. 2921–2929. https://doi.org/10.1109/CVPR.2016.319.

40. Sundararajan, M., Taly, A., and Yan, Q. (2017). Axiomatic Attribution for Deep Networks. In 34th Int. Conf. Mach. Learn., *7* (ICML), pp. 5109–5118.

STAR★METHODS

KEY RESOURCES TABLE

REAGENT or RESOURCE	SOURCE	IDENTIFIER
Deposited data		
Panoptes implemented with TensorFlow v2		https://github.com/Wenke-Liu/panoptes
Panoptes implemented with TensorFlow v1	Hong et al.[5]	https://github.com/rhong3/CPTAC-UCEC
Genomic Data Commons Data Portal	National Cancer Institute	https://portal.gdc.cancer.gov
TCIA[33]	The Cancer Imaging Archive[33]	https://www.cancerimagingarchive.net/collections
IDC	Imaging Data Commons	https://portal.imaging.datacommons.cancer.gov/explore/
CPTAC Pan-Cancer Proteogenomics Data	Li et al.[22]	https://www.cell.com/cancer-cell/fulltext/S1535-6108(23)00219-2
Software and algorithms		
Panoptes Python3 package	Hong et al.[5]	https://pypi.org/project/panoptes-he/
TensorFlow	Abadi et al.[34]	https://www.tensorflow.org
Inception	Szegedy et al.[35]	https://github.com/google/inception
InceptionResNet	Szegedy et al.[36]	https://github.com/tensorflow/models/tree/master/research/slim/nets
Keras	Chollet et al.[37]	https://keras.io
Other		
NVIDIA Tesla V100 GPU	NYU Langone Health BigPurple HPC Cluster	http://bigpurple-ws.nyumc.org/wiki/index.php/BigPurple_HPC_Cluster
NVIDIA Tesla P40 GPU	NYU HPC Cluster	https://wikis.nyu.edu/display/NYUHPC/High+Performance+Computing+at+NYU

RESOURCE AVAILABILITY

Lead contact

Further information and requests for resources should be directed to and will be fulfilled by the Lead Contact, David Fenyö (David@FenyoLab.org).

Materials availability

This study did not generate new unique reagents.

Data and code availability

- Digitized H&E slides from CPTAC are publicly available at The Cancer Imaging Archive (TCIA).[33] Clinical data, demographics and other clinical features of these patients were extracted from tissue-specific CPTAC studies, and harmonized by the CPTAC Pan-Cancer Proteogenomics consortium. Full details appear in the Companion Pan-Cancer Resource manuscript.[22] We focused on the CPTAC samples with genomic, transcriptomic, and proteomic data available to investigate the pan-cancer proteogenomic impacts on histopathology.
- Raw and processed proteomics as well as open access genomic data can be obtained via Proteomic Data Commons (PDC) at https://pdc.cancer.gov/pdc/cptac-pancancer. Raw genomic and transcriptomic data files can be accessed via the Genomic Data Commons (GDC) Data Portal at https://portal.gdc.cancer.gov with dbGaP Study Accession: phs001287.v16.p6. Complete CPTAC pan-cancer controlled and processed data can be accessed via the Cancer Data Service (CDS, https://dataservice.datacommons.cancer.gov/). The CPTAC pan-cancer data hosted in CDS is controlled data and can be accessed through the NCI DAC approved, dbGaP compiled whitelists. Users can access the data for analysis through the Seven Bridges Cancer Genomics Cloud (SB-CGC) which is one of the NCI-funded Cloud Resource/platform for compute intensive analysis.

Instructions to access data.

1. Create an account on CGC, Seven Bridges (https://cgc-accounts.sbgenomics.com/auth/register)
2. Get approval from dbGaP to access the controlled study (https://www.ncbi.nlm.nih.gov/projects/gap/cgi-bin/study.cgi?study_id=phs001287.v16.p6)

CellPress
OPEN ACCESS

3. Log into CGC to access Cancer Data Service (CDS) File Explore
4. Copy data into your own space and start analysis and exploration
5. Visit the CDS page on CGC to see what studies are available and instructions and guides to use the resources. (https://docs.cancergenomicscloud.org/page/cds-data)

- All original code has been deposited at GitHub and is publicly available as of the date of publication. Links are listed in the key resources table.
- Any additional information required to reanalyze the data reported in this work paper is available from the lead contact upon request.

EXPERIMENTAL MODEL AND SUBJECT DETAILS

The study was entirely computational and did not involve human subjects as it obtained neither data through intervention or interaction with living individuals nor identifiable private information.

METHOD DETAILS

Image and data acquisition

Images consisted of control diagnostic H&E-stained slides sectioned from formalin-fixed paraffin-embedded (FFPE) tissue blocks obtained from the Clinical Proteomic Tumor Analysis Consortium (CPTAC), representing mirror-image sections matched to the cognate OCT embedded tissue samples used for molecular analyses. These samples covered six cancer types: Clear Cell Renal Cell Carcinoma (CCRCC), Head and Neck Squamous Cell Carcinoma (HNSCC), Lung Squamous Cell Carcinoma (LSCC), Lung Adenocarcinoma (LUAD), Pancreatic Ductal Adenocarcinoma (PDA), and Uterine Corpus Endometrial Carcinoma (UCEC) with 2217 total tumor tissue slides and 538 normal adjacent tissue (NAT) slides from 657 patients (Figure S1A). Clinical features, histopathological features, and gene mutation information for these samples were obtained from CPTAC (Figures S1B and S1C). Expression data for RNA-Seq and proteomics was processed and provided from the CPTAC Pan-Cancer proteogenomic dataset.[22] In summary, RNA and proteome expression was collected individually from each of the respective flagship manuscripts corresponding to each disease tissue, and harmonized with tissue-level batch correction. Each whole slide image is divided into smaller "tiles" sharing the same label assigned to that slide. Panoptes-based multi-resolution CNN models were then trained, validated, and tested on the tiles. Tile-level probabilities and latent features are mean-aggregated for slide-level evaluation. To ensure that the models captured pan-cancer level features rather than tumor type-specific ones, each sample weight was inversely proportional to both its cancer type prevalence and frequency of its class label. This approach encourages models to more heavily penalize incorrect predictions of images from under-represented groups and "pay more attention" to those images. Cancer type-specific models were also trained and cross-tested by comparing features and performance with that of the pan-cancer models.

H&E slide processing and sample preparation

Each histopathology image was scanned at a maximum depth of 20x resolution. Digital histopathologic images were in SVS or SCN format, which were tuples of the same images at multiple different resolutions. They were segmented into smaller tiles of 299 by 299 pixels with an overlapping area of 49 pixels between each tile. Tiles were grouped at the 10x, 5x, and 2.5x resolutions and geographically linked such that the model always viewed tiles in the same spatial region. Tiles that contained artifacts due to poor scanning, previous annotations marked by pathologists, and excess white space (>60%) were removed prior to training. To account for differences in staining procedures by different institutions, Vahadane's color normalization was applied.[38] Images were split at the patient-level into training, validation, and test sets with a 70:15:15 ratio using stratified sampling. Images from the same patient were always confined to the same set.

Computational method of deep learning models

Panoptes1 architecture, which is a multiresolutional architecture based on InceptionResnet1, was trained into deep learning imaging models for this study. All the models were trained with randomly initialized network parameters with auxiliary classifiers opened on each branch. Tiles of 10x, 5x, and 2.5x resolutions of the same region on the H&E slide with label were paired and considered as 1 sample as only 1 prediction score was associated with a multi-resolution matrix. Softmax cross entropy loss was weighed by training data composition and tumor types, and Adam optimization algorithm was applied in the training workflow. Batch size was set to 24, which was the largest number that could fit in the memory of our GPUs, and the initial learning rate was set to 0.0001 with a drop-out rate of 0.5.100 batches of validation were carried out every 1000 iterations of training and when the training loss achieved a new minimum value after 30000 iterations of training. If the mean of these 100-batch validation loss achieved minimum, the model was saved as the temporary best performing model. The training process stopped when the validation loss did not decrease for at least 10000 iterations. This stopping criterion was only initiated after 100000 iterations of training.

Feature extraction and visualization

The feature maps before the last fully connected layer in the model of the test set samples were exported, in which each sample is represented as a 1-dimensional vector, together with their prediction scores and true labels. We then used tSNE with initial dimensions of 100 to reduce these vectors into 2-dimensional space where each point represents a sample. Generally, points clustered according to their predicted class. By replacing the points on tSNE plots with the original tiles, the features learned by the model for each of the specific classes can be observed. We asked experienced pathologists to summarize the typical histological features in each of these clusters. We also applied the vanilla class activation mapping (CAM)[39] to project the classification weights back onto the activations of the test set samples and rescaled them to 299 × 299 pixel images, which were then aggregated back to intact slide size. The slide level CAM visualizations were then overlaid to the original slides to demonstrate the potential focuses of the models. In addition, we applied saliency maps technique using the Saliency package[40] in Python3. Here, the gradients were back-propagated to the input layer, which was then overlaid to the original tiles. Similar to CAM, they were aggregated to the slide-level to illustrate the potential feature importance used by the models. We also used the prediction scores of tiles directly to build slide-level prediction heatmaps, which showed the heterogeneity of prediction results of tumor slides.

Omics integration with sparse multiCCA

Traditional CCA only works with two dataset inputs (*X*, *Y*) and seeks to find two canonical variates (α, β) such that $Cor(X\alpha_i, Y\beta_i)$ is maximized for each *i*-th component. In the case of high-dimensional data (as is common for *omics*), the covariance matrix is invertible, and so a CCA solution cannot be calculated. This high-dimensionality problem was addressed with a sparse CCA method[35] by incorporating penalty parameters such that the objective function changes to maximizing $Cor(X\alpha_i, Y\beta_i) - P_1(\alpha) - P_2(\beta)$ where $P_1(\alpha)$ and $P_2(\beta)$ represent penalty functions on the two canonical variates. Multiple sparse CCA expands this approach to incorporate a third dataset input (*X, Y, Z*) to calculate three canonical variates (α, β, γ) where $Cor(X\alpha_i, Y\beta_i, Z\gamma_i) - P_1(\alpha) - P_2(\beta) - P_3(\gamma)$ is maximized. Columns of image features (*X*), transcriptomic (*Y*) and proteomic (*Z*) matrices will be standardized to mean zero and unit variance prior to computation. The objective function is iterated to identify *i* components, where $i = 100$.

To reduce overfitting, we applied sparse multiCCA using the R package PMA for latent imaging features (*X*), RNA-Seq expression (*Y*) and proteome expression (*Z*) only with samples designated in the testing set. Genes with no expression in 60% or more of all samples were filtered out. Three penalty parameters for each matrix were established through 10-fold cross validation and a heatmap of the grid search illustrates the average pairwise correlations (*X* and *Y*, *X* and *Z*, *Y* and *Z*) from each of the first components within each task. Only models with satisfactory AUC performance (0.75) were assessed. In addition, we determined the correlation from randomly shuffling the rows of *X*, *Y*, and *Z* such that the pairwise association between samples is removed but the biological signal within each sample is retained. Each of these permutations was randomized in a consistent manner across different penalty parameters and tasks with a seed. In combination, these permutations validate the extent to which signals are captured by sparse multiCCA. Consistently across tasks, the test correlation significantly exceeds the permuted correlations.

To identify the subset of genomic networks regulating morphology phenotypes, we aim to select sparsity parameters that maximize the correlation from cross-fold validation, and also produce small numbers of genes and protein features within each component. With this objective, the penalty parameter for *X* that nets the highest average correlation is first fixed, followed by parameters for *Y* and *Z*. Using these parameters, we re-assessed the extent to which random associations may be extracted by sparse multiCCA with the random permutations technique described previously. The average correlation of the first 50 components from both the non-permuted and permuted sets show significant differences (Figure S4), suggesting that sparse multiCCA has captured genuine signals of potential biological interest. Gene and protein sets were tested for enriched gene ontology (GO) terms using clusterProfiler and org.Hs.eg.db R packages. Within each component, images and tiles with the highest and lowest scores were selected for visualization.

Minimizing effects of tissue of origin on pan-cancer mutation prediction

We used weighted loss inversely proportional to the cancer types in order to minimize the effects of tissue origin as confounding factors and ensure that the models learned features related to mutation status independent of cancer type. One-tail Wilcoxon tests on the prediction scores between mutated and wild-type samples (labeled positive and negative respectively) were conducted at the per-tile level.

Cell Reports Medicine, Volume 4

Supplemental information

Deep learning integrates histopathology and proteogenomics at a pan-cancer level

Joshua M. Wang, Runyu Hong, Elizabeth G. Demicco, Jimin Tan, Rossana Lazcano, Andre L. Moreira, Yize Li, Anna Calinawan, Narges Razavian, Tobias Schraink, Michael A. Gillette, Gilbert S. Omenn, Eunkyung An, Henry Rodriguez, Aristotelis Tsirigos, Kelly V. Ruggles, Li Ding, Ana I. Robles, D.R. Mani, Karin D. Rodland, Alexander J. Lazar, Wenke Liu, David Fenyö, and Clinical Proteomic Tumor Analysis Consortium

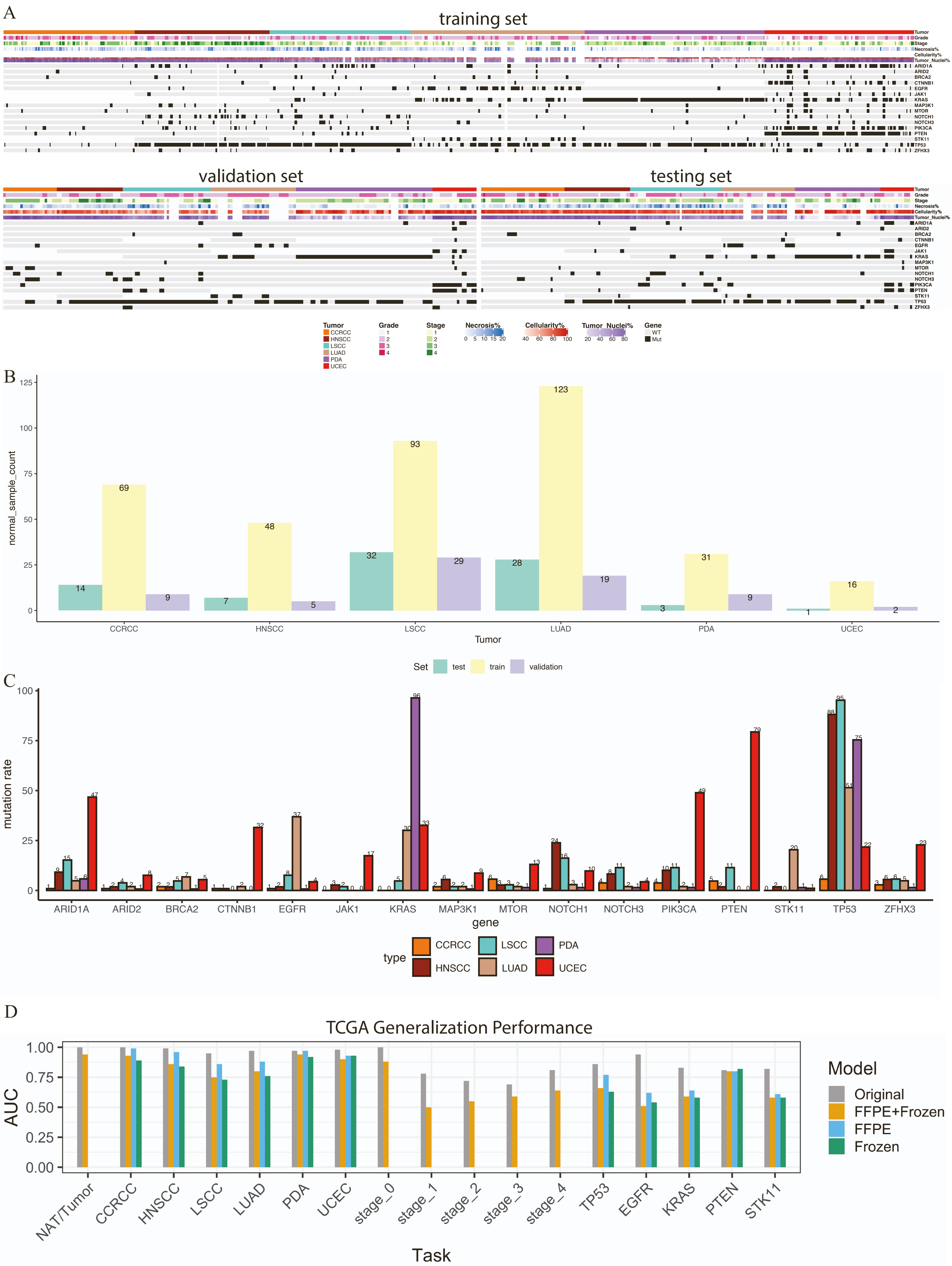
A
training set
validation set
testing set
Tumor
Grade
Stage
Necrosis%
Cellularity%
Tumor_Nuclei%
ARID1A
ARID2
BRCA2
CTNNB1
EGFR
JAK1
KRAS
MAP3K1
MTOR
NOTCH1
NOTCH3
PIK3CA
PTEN
STK11
TP53
ZFHX3
Tumor
CCRCC
HNSCC
LSCC
LUAD
PDA
UCEC
Grade
1
2
3
4
Stage
1
2
3
4
Necrosis%
0 5 10 15 20
Cellularity%
40 60 80 100
Tumor_Nuclei%
20 40 60 80
Gene
WT
Mut
B
normal_sample_count
Tumor
CCRCC
HNSCC
LSCC
LUAD
PDA
UCEC
Set
test
train
validation
C
mutation rate
gene
ARID1A
ARID2
BRCA2
CTNNB1
EGFR
JAK1
KRAS
MAP3K1
MTOR
NOTCH1
NOTCH3
PIK3CA
PTEN
STK11
TP53
ZFHX3
type
CCRCC
HNSCC
LSCC
LUAD
PDA
UCEC
D
TCGA Generalization Performance
AUC
Task
NAT/Tumor
CCRCC
HNSCC
LSCC
LUAD
PDA
UCEC
stage_0
stage_1
stage_2
stage_3
stage_4
TP53
EGFR
KRAS
PTEN
STK11
Model
Original
FFPE+Frozen
FFPE
Frozen

Figure S1: Data summary and generalization performance, related to Figure 1.

(A) Heatmaps show clinical and molecular features with 70:15:15 split at per-patient level for training, validation and testing sets.

(B) Number of normal slides in each set.

(C) Common gene mutation rates of samples in our cohort by cancer type.

(D) TCGA external validation for all imaging-based prediction tasks reported at per-slide AUROCs.

A

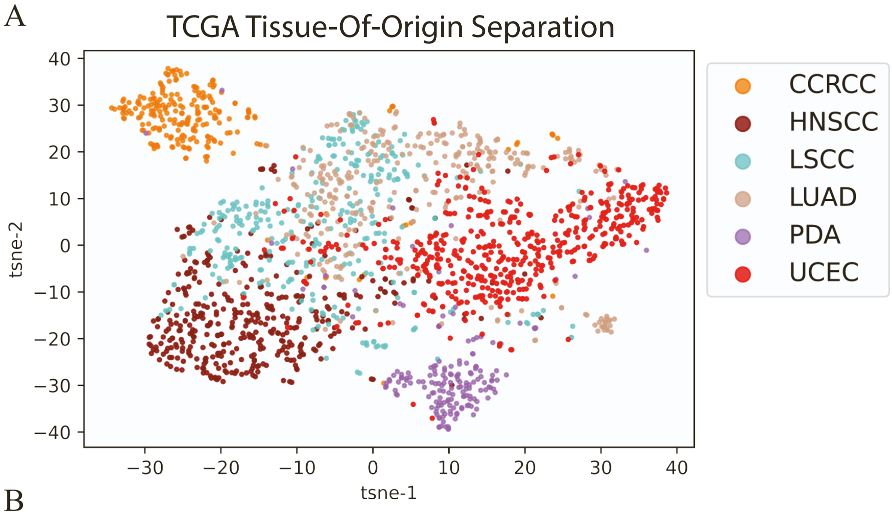

B

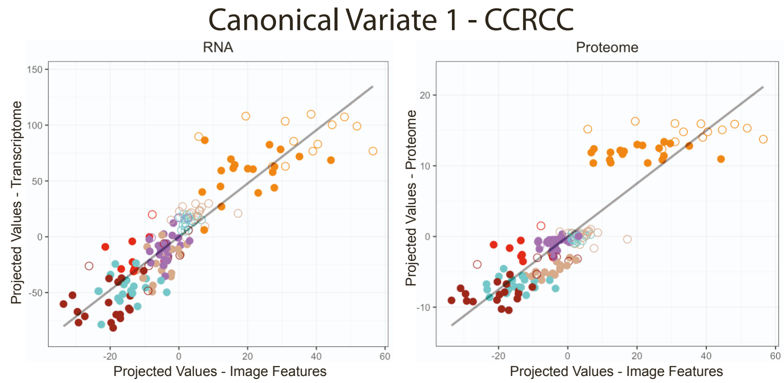

top GO terms (RNA)	q
fatty acid oxidation	1.5 e-18
alpha-amino acid catabolic process	6.9 e-11
peroxisome organization	1.6 e-10

bottom GO terms (RNA)	q
regulation of chromosome segregation	4.1 e-13
cornification	1.1 e-10
keratinization	1.2 e-10

top GO terms (Proteome)	q
aromatic amino acid family catabolic process	1.0 e-2
endothelium development	1.1 e-2
actin filament capping	1.1 e-2

bottom GO terms (Proteome)	q
epidermis morphogenesis	8.8 e-4
regulation of hair cycle	1.1 e-3
intermediate filament organization	1.2 e-3

C

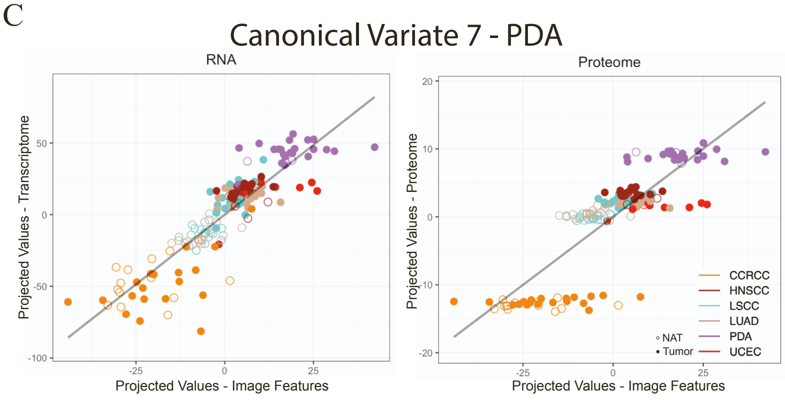

top GO terms (RNA)	q
collagen metabolic process	1.5 e-6
SRP-dependent cotranslational protein targeting to membrane	4.6 e-6
extracellular matrix disassembly	1.8 e-4

bottom GO terms (RNA)	q
alpha-amino acid catabolic process	4.4 e-9
dicarboxylic acid metabolic process	1.5 e-7
cofactor catabolic process	2.5 e-4

top GO terms (Proteome)	q
regulation of epithelial cell apoptotic process	4.4 e-3
regulation of response to wounding	4.7 e-3
negative regulation of Wnt signaling pathway	4.7 e-3

D

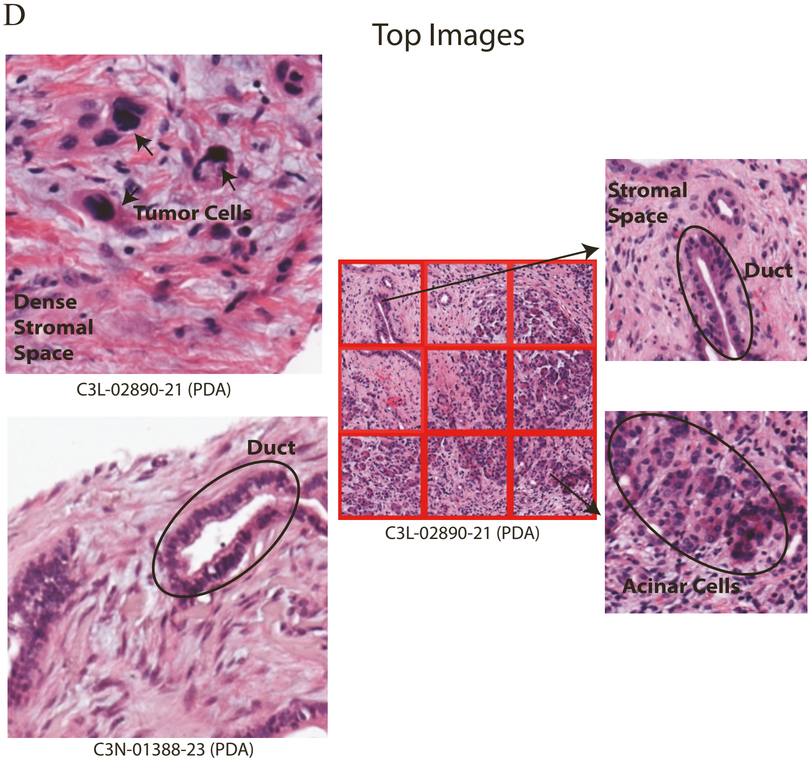

Figure S2: Additional covariates explaining tissue-of-origin imaging features, related to Figure 2.

(A) TSNE separation of latent features from external TCGA test set.

(B) Strongest covariate distinctly separates CCRCC from all others. Top and bottom GO terms explain the enrichment of genes/proteins selected to separate images into top and bottom scores respectively.

(C) Top terms correspond to enrichment of genes/proteins separating PDA samples from others.

(D) Top scoring images exhibit morphologies consistent with selected proteogenomic features.

A

B

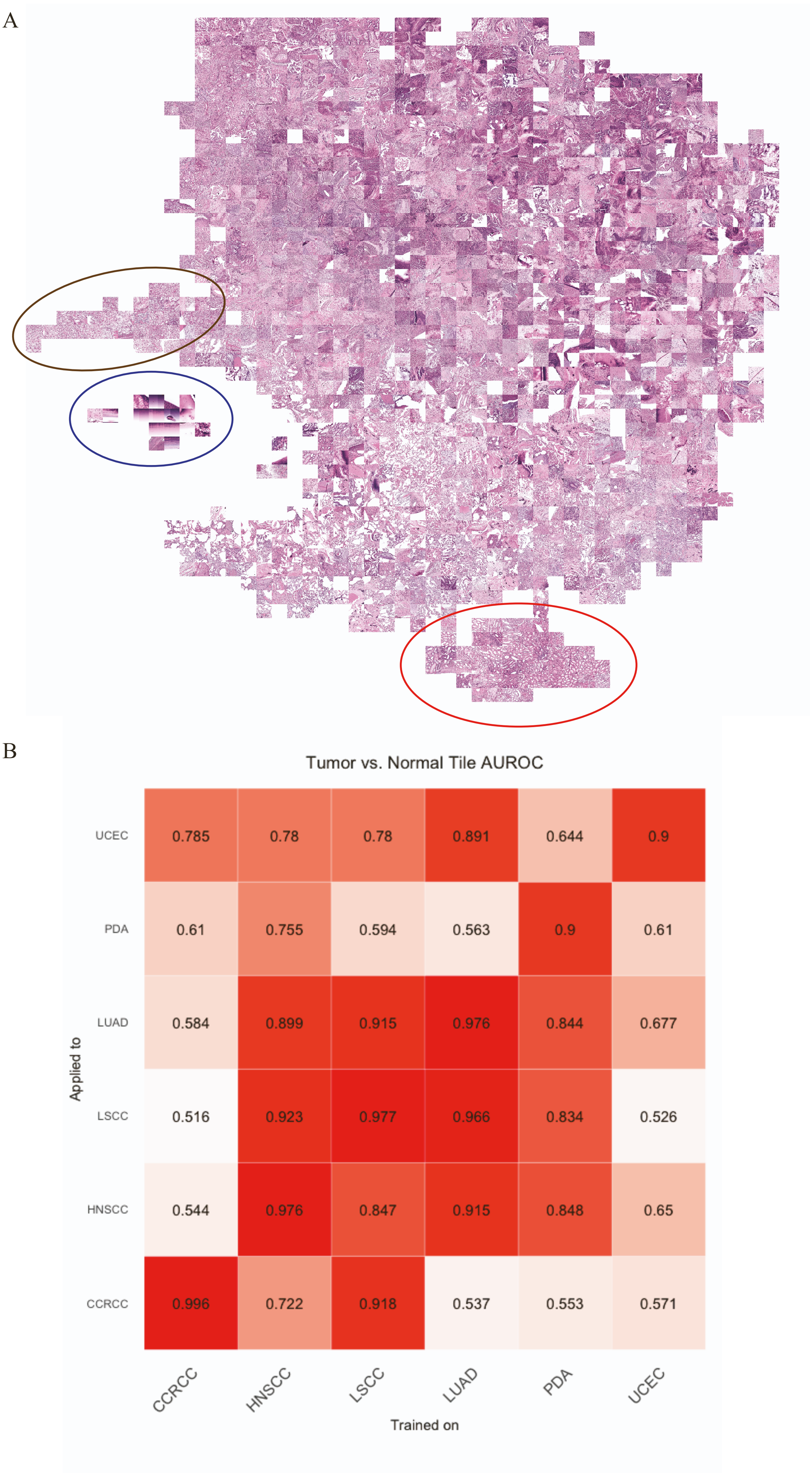

Tumor vs. Normal Tile AUROC
UCEC
PDA
LUAD
LSCC
HNSCC
CCRCC
Applied to
0.785
0.78
0.78
0.891
0.644
0.9
0.61
0.755
0.594
0.563
0.9
0.61
0.584
0.899
0.915
0.976
0.844
0.677
0.516
0.923
0.977
0.966
0.834
0.526
0.544
0.976
0.847
0.915
0.848
0.65
0.996
0.722
0.918
0.537
0.553
0.571
CCRCC
HNSCC
LSCC
LUAD
PDA
UCEC
Trained on

Figure S3: Detailed feature visualization of tumorigenesis model and per-tile level cross testing performance, related to Figure 3.

(A) Extraction of features learned by pan-cancer tumorigenesis model. Brown circle highlighting distinctive CCRCC features. Red circle highlighting non-neoplastic renal tubules. Blue circle highlighting artifacts.

(B) Heatmap showing the per-tile level AUROCs of applying single cancer type trained models to the other cancer type samples.

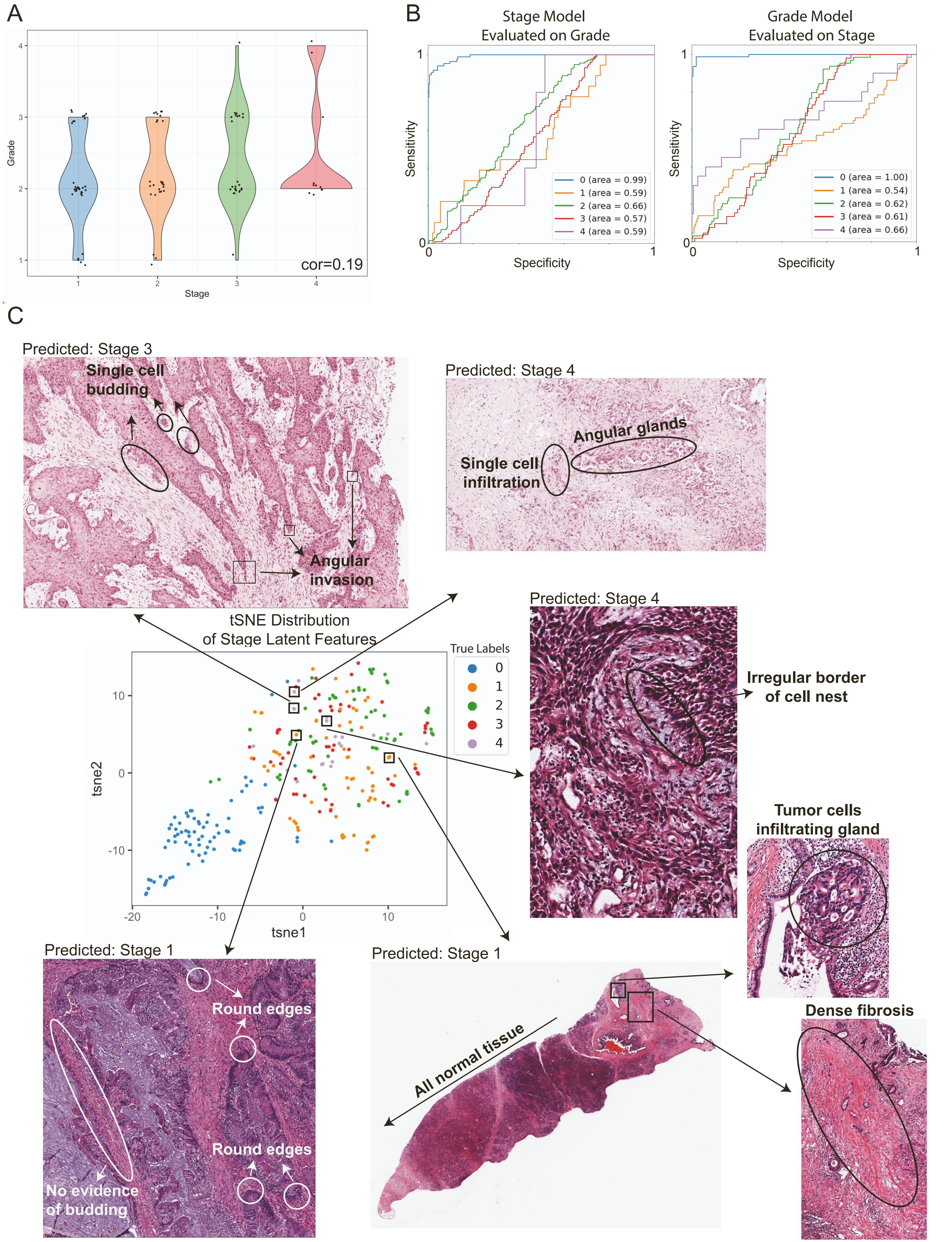
A
Grade
Stage
cor=0.19
B
Stage Model
Evaluated on Grade
Sensitivity
Specificity
0 (area = 0.99)
1 (area = 0.59)
2 (area = 0.66)
3 (area = 0.57)
4 (area = 0.59)
Grade Model
Evaluated on Stage
Sensitivity
Specificity
0 (area = 1.00)
1 (area = 0.54)
2 (area = 0.62)
3 (area = 0.61)
4 (area = 0.66)
C
Predicted: Stage 3
Single cell budding
Angular invasion
Predicted: Stage 4
Angular glands
Single cell infiltration
tSNE Distribution
of Stage Latent Features
True Labels
tsne1
tsne2
Predicted: Stage 4
Irregular border of cell nest
Tumor cells infiltrating gland
Predicted: Stage 1
Round edges
Round edges
No evidence of budding
Predicted: Stage 1
All normal tissue
Dense fibrosis

Figure S4: Slide level performance of percent nuclei, necrosis, and cellularity, related to Figure 5.

(A) CPTAC grade and stage labels do not correlate.
(B) Models trained to predict grade and stage do not generalize to predicting the other label.
(C) Manual histology review of differing features amongst samples predicted at varying degrees of tumor stage.

A

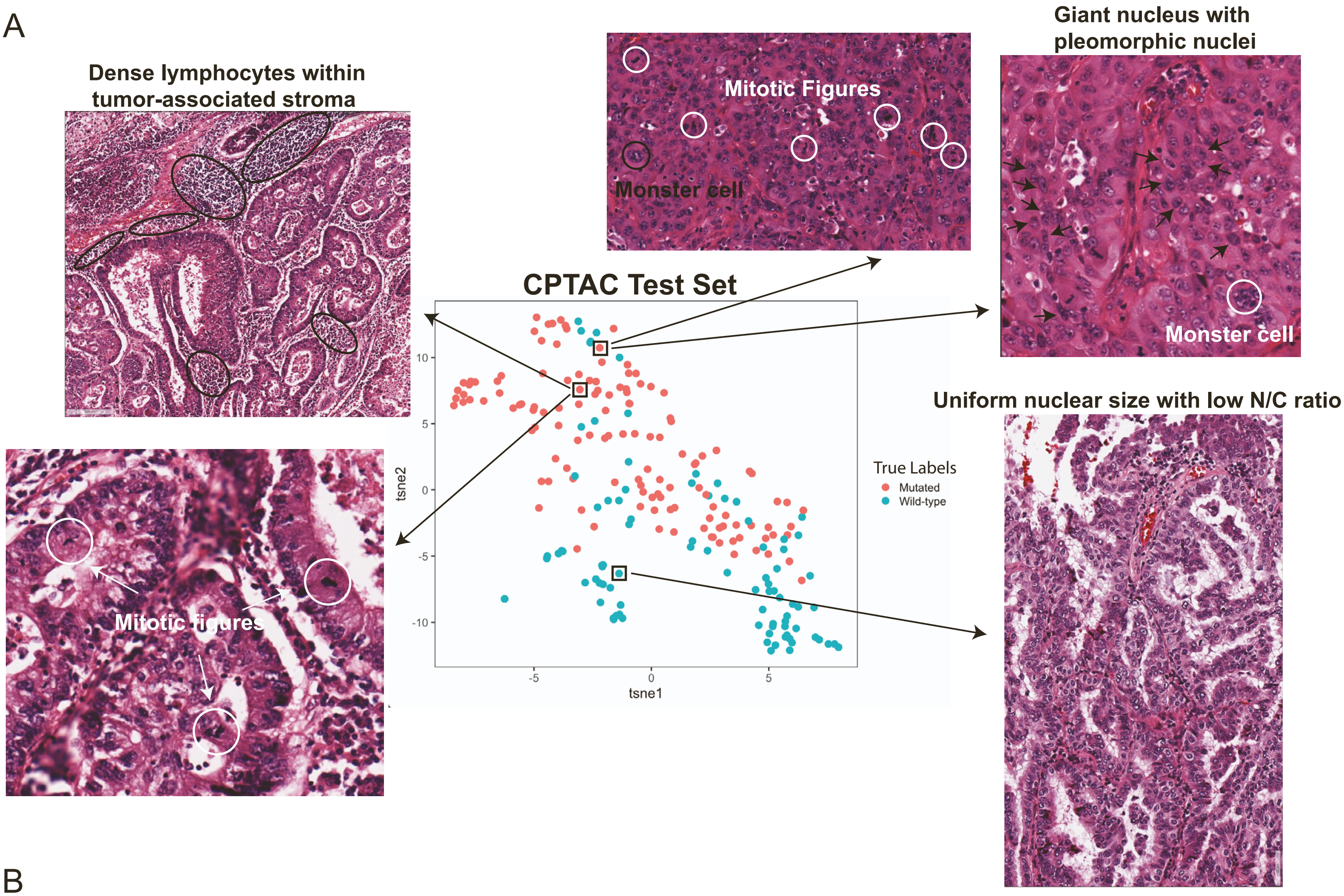
Dense lymphocytes within tumor-associated stroma
Mitotic Figures
Monster cell
Giant nucleus with pleomorphic nuclei
Monster cell
CPTAC Test Set
tsne2
tsne1
True Labels
Mutated
Wild-type
Uniform nuclear size with low N/C ratio
Mitotic figures

B

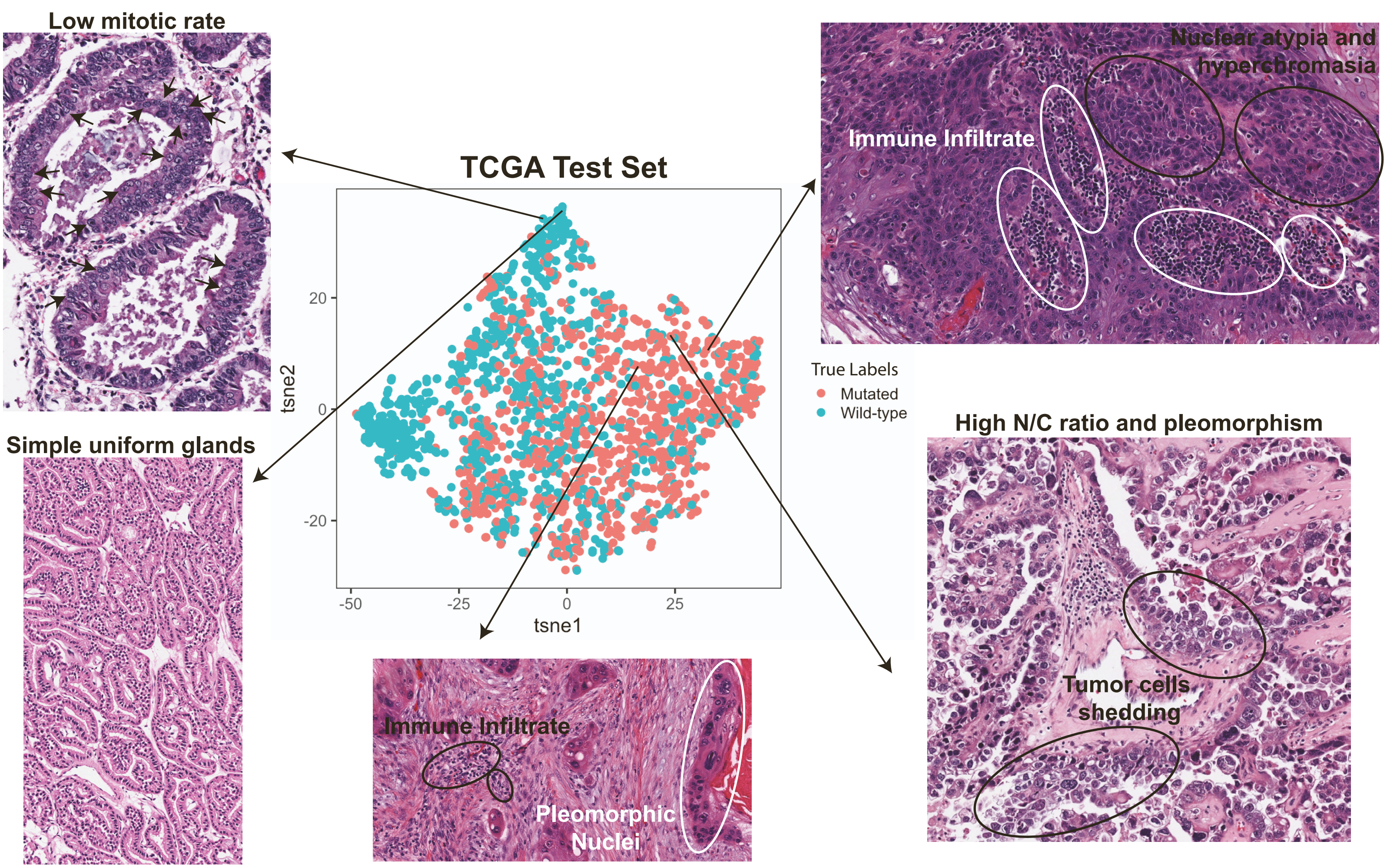
Low mitotic rate
Nuclear atypia and hyperchromasia
Immune Infiltrate
TCGA Test Set
tsne2
tsne1
True Labels
Mutated
Wild-type
Simple uniform glands
High N/C ratio and pleomorphism
Tumor cells shedding
Immune Infiltrate
Pleomorphic Nuclei

Figure S5: Visualization of features learned by the TP53 model for internal and external test sets, related to Figure 6.

(A) CPTAC.

(B) TCGA.

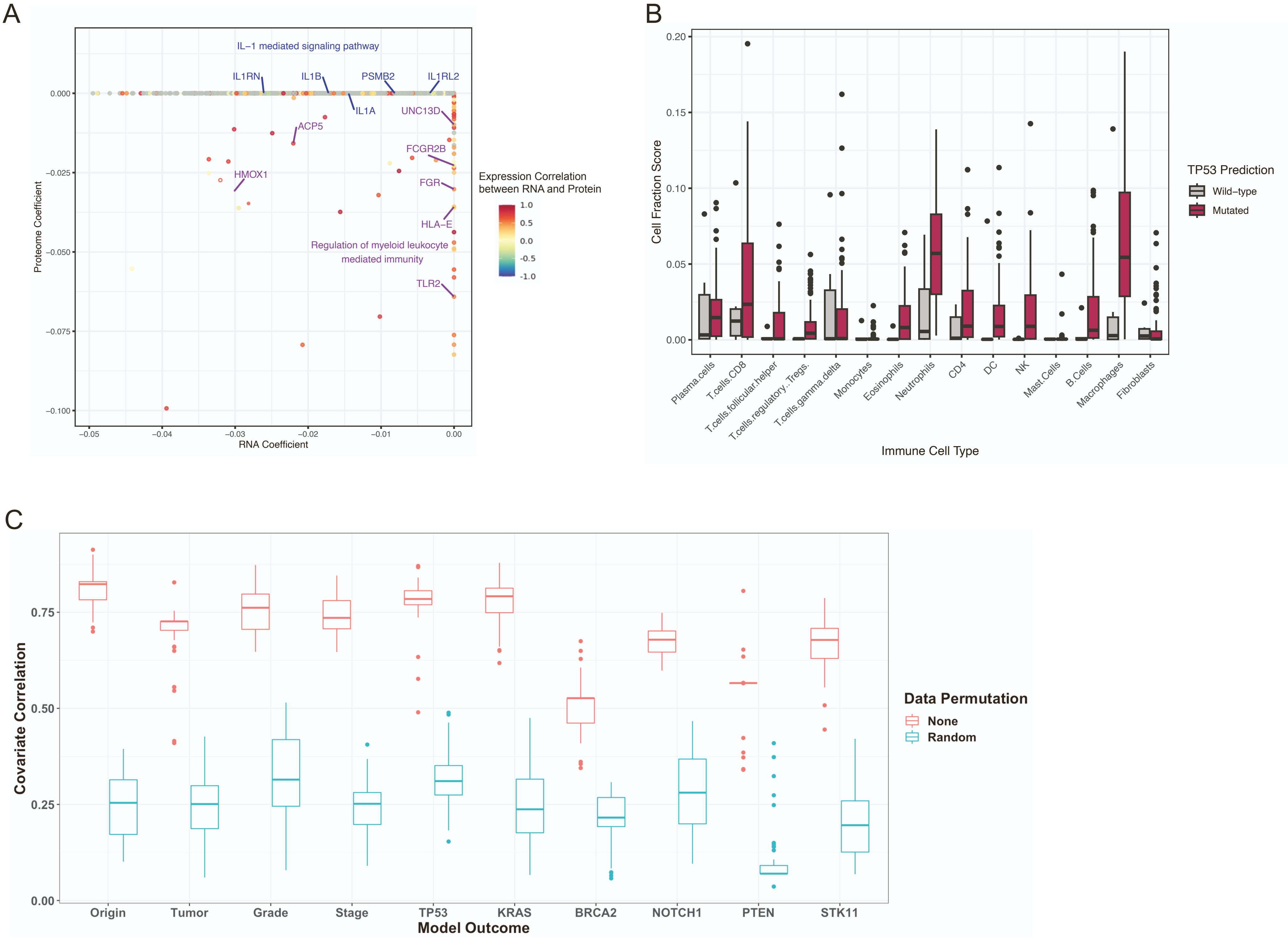
A
IL−1 mediated signaling pathway
IL1RN
IL1B
PSMB2
IL1RL2
IL1A
UNC13D
ACP5
FCGR2B
HMOX1
FGR
HLA−E
Regulation of myeloid leukocyte mediated immunity
TLR2
Proteome Coefficient
RNA Coefficient
0.000
−0.025
−0.050
−0.075
−0.100
−0.05
−0.04
−0.03
−0.02
−0.01
0.00
Expression Correlation between RNA and Protein
1.0
0.5
0.0
-0.5
-1.0
B
Cell Fraction Score
0.20
0.15
0.10
0.05
0.00
Plasma.cells
T.cells.CD8
T.cells.follicular.helper
T.cells.regulatory..Tregs.
T.cells.gamma.delta
Monocytes
Eosinophils
Neutrophils
CD4
DC
NK
Mast.Cells
B.Cells
Macrophages
Fibroblasts
Immune Cell Type
TP53 Prediction
Wild−type
Mutated
C
Covariate Correlation
0.75
0.50
0.25
0.00
Origin
Tumor
Grade
Stage
TP53
KRAS
BRCA2
NOTCH1
PTEN
STK11
Model Outcome
Data Permutation
None
Random

Figure S6: Paired imaging-proteogenomic were randomly permuted to assess the baseline correlation detectable due to noise, related to Figure 6.

(A) Plot of the coefficients of each gene at the RNA and protein level. The dot color represents the expression correlation at RNA and protein level. Genes selected as important at the RNA level but not proteome will have a zero value on the y-axis but a non-zero value on the x-axis, and vice versa for those correlated at the proteome level but not RNA.

(B) Boxplot distribution of immune cell fractions calculated from BayesDeBulk proteogenomic deconvolution in TP53-mutated and wildtype test samples.

(C) Across all model outcomes, canonical variate correlations are significantly higher than that from permutation, suggesting that the additional correlation captured corresponds to genuine biological signals.
